# A mouse model of miR-96, miR-182 and miR-183 misexpression implicates miRNAs in cochlear cell fate and homeostasis

**DOI:** 10.1038/s41598-018-21811-1

**Published:** 2018-02-23

**Authors:** Michael D. Weston, Shikha Tarang, Marsha L. Pierce, Umesh Pyakurel, Sonia M. Rocha-Sanchez, JoAnn McGee, Edward J. Walsh, Garrett A. Soukup

**Affiliations:** 1 Department of Oral Biology, School of Dentistry, Creighton University, 780729 California Plaza, Omaha, NE 68178-0729 USA; 2 Department of Pharmacology, School of Medicine, Creighton University, 2500 California Plaza, Omaha, NE 68178 USA; 3 Developmental Auditory Physiology Laboratory, Boys Town National Research Hospital, 555 North 30th Street, Omaha, NE 68131 USA; 4 Department of Biomedical Sciences, School of Medicine, Creighton University, 2500 California Plaza, Omaha, NE 68178 USA

## Abstract

Germline mutations in *Mir96*, one of three co-expressed polycistronic miRNA genes (*Mir96, Mir182, Mir183*), cause hereditary hearing loss in humans and mice. Transgenic FVB/NCrl- Tg(GFAP-*Mir183,Mir96,Mir182*)MDW1 mice (Tg^1MDW^), which overexpress this neurosensory-specific miRNA cluster in the inner ear, were developed as a model system to identify, in the aggregate, target genes and biologic processes regulated by the miR-183 cluster. Histological assessments demonstrate Tg^1MDW/1MDW^ homozygotes have a modest increase in cochlear inner hair cells (IHCs). Affymetrix mRNA microarray data analysis revealed that downregulated genes in P5 Tg^1MDW/1MDW^ cochlea are statistically enriched for evolutionarily conserved predicted miR-96, miR-182 or miR-183 target sites. ABR and DPOAE tests from 18 days to 3 months of age revealed that Tg^1MDW/1MDW^ homozygotes develop progressive neurosensory hearing loss that correlates with histologic assessments showing massive losses of both IHCs and outer hair cells (OHCs). This mammalian miRNA misexpression model demonstrates a potency and specificity of cochlear homeostasis for one of the dozens of endogenously co-expressed, evolutionally conserved, small non-protein coding miRNA families. It should be a valuable tool to predict and elucidate miRNA-regulated genes and integrated functional gene expression networks that significantly influence neurosensory cell differentiation, maturation and homeostasis.

## Introduction

MicroRNAs are short (~20–24nt) endogenous non-coding RNAs that posttranscriptionally repress the expression of protein-coding genes. The effects mediated by miRNAs on inner ear morphogenesis, neurosensory cell identity, function and homeostasis indicate that gene regulation through miRNAs are critical to the biology of the inner ear^1–6^. The polycistronic cluster of *Mir96*, *Mir182* and *Mir183* genes are abundantly expressed in afferent cochlear and vestibular neurons and their peripheral innervating targets: auditory and vestibular hair cells (HCs)^7–9^. From an evolutionary viewpoint, the miR-183 cluster of miRNA genes are syntenic, highly conserved and co-expressed in neurosensory organs of animals representing several taxonomic phyla, suggesting that the control of gene expression through by this miRNA cluster is highly coordinated and under extraordinary selective pressure^10^.

The spatiotemporal expression pattern of *Mir96*, *Mir182* and *Mir183* in the developing vertebrate inner ear and the effects induced by modulating levels of these miRNAs on HC fate determination in zebrafish and chicken argue that these miRNAs collectively function, to some degree, in the transition from inner ear prosensory cells towards a HC fate^8,9,11,12^. Also, they have significant roles in HC functional maturation and homeostasis^2,5,13,14^. Genome-wide studies demonstrate that miRNAs can fine-tune mRNA levels in the context of miRNA/target mRNA co-expression and can also act as developmental on-off switches, as in the context of mutually exclusive miRNA/target mRNA expression^15–19^. For those effects attributable to the miR-183 cluster in the vertebrate inner ear, these functional distinctions in miRNA regulation might be segregated temporally: in a switch-like manner in the case of HC cell fate assignment, then in a fine-tuning manner in the case of HC morpho-functional maturation and homeostasis.

During development of the cochlear neurosensory epithelium (i.e. organ of Corti or OC), the prosensory cell lineage differentiates as either HC or supporting cells (SCs), a process mediated in part through Delta-Notch signaling^20^. By E15.5, miR-183 cluster expression distinguishes differentiated cochlear HC, which suggests a role for these miRNAs in this cell type transition^9^. Based on these data, we hypothesized that the miR-183 cluster, if misexpressed in SCs, would, in the context of mutual exclusion, perturb SC gene expression. The observation of aggregate effects would help establish evolutionarily conserved miRNA function(s) with potential relevance with regard to the treatment of hearing loss through HC regeneration strategies^6^. To test this hypothesis, we engineered Tg(GFAP-*Mir183,Mir96,Mir182*) (Tg^1MDW^) mice to drive ectopic miR-183 cluster expression using the core human promoter of the glial fibrillary acidic protein (GFAP). Overall, Tg^1MDW^ cochlear HC and SC differentiate and histologically mature in a similar pattern to that of wild-type littermates. Nonetheless, HC lifespan is significantly shortened and hearing function is rapidly lost: most cochlear HCs are lost by the time the mice are 6 months old, resulting in severe deafness. These data are consistent with the view that HC microRNA misexpression in lineage-related SCs has effects on their capacity to maintain HC homeostasis. Furthermore, the gene expression effects, in aggregate and in identified gene ontology pathways, provide basic information necessary to gain a more complete understanding not only of the role these microRNAs serve during HC maturation, but also those genes and processes that are particularly sensitive to SC miRNA misexpression.

**Table 1:** Tg-(GFAP-*Mir183,Mir96,Mir182*) line information.

Line name	FISH localization	Intercross genotypes scored	Mendelian ratio χ^2^ P-value	Tg genotype (log2^−∆∆CT^)	MGI submission #
		WT = 154		N/A	
Tg^1MDW^	Chr 9E3	Het = 240	0.017	5.9 ± 1.3	MGI:5436579
		Homo = 113		10.6 ± 2.4	
		WT = 23		N/A	
Tg^2MDW^	Chr 16C1~3.1	Het = 36	0.40	7.5 ± 2.8	MGI:5436582
		Homo = 25		15.6 ± 6.7	
		WT = 25		N/A	
Tg^3MDW^	Chr 16 A~B2	Het = 49	0.98	2.2 ± 0.3	MGI:5436584
		Homo = 22		3.9 ± 0.7	

## Results

### FVB/NClr-Tg(GFAP-*Mir183,Mir96,Mir182*)1MDW mice

SCs in the postnatal inner ear organ of Corti (OC) express endogenous GFAP and human GFAP-promoter driven GFP and LacZ reporters^21,22^. To direct misexpression of miR-96, miR-182 and miR-183 in the SCs of the inner ear, we modified an established GFAP promoter-driven reporter construct (pGFA-nlac, Michael Brenner, UAB) by substituting the nLacZ gene with the miR-183 cluster coding sequences^23^. Of 425 implanted embryos, 3 of the 16 live born pups were identified as founders and three independent inbred lines were established in FVB/NClr: Tg(GFAP-*Mir183,Mir96,Mir182*)1MDW, Tg(GFAP-*Mir183,Mir96,Mir182*)2MDW, Tg(GFAP-*Mir183,Mir96,Mir182*)3MDW (Tg^1MDW^, Tg^2MDW^, Tg^3MDW^). All lines transmit at single-gene Mendelian ratios and map to unique cytogenetic loci (Table 1). At a gross phenotypic level, adult homozygous Tg^1MDW/1MDW^ mice lose Preyer’s reflex, an indication of hearing loss^24^. Both homozygous and hemizygous mice from Lines Tg^1MDW^ and Tg^2MDW^ develop lens cataracts with average onset ages, in days, of 95 ± 11 n = 127, 121 ± 23 n = 19, 132 ± 17 n = 12, 157 ± 5 n = 14, respectively (Kruskal–Wallis P < 0.0001). The Tg^3MDW^ homozygous and hemizygous mice exhibit a progressive loss of pelage clearly evident by P50 that progresses steadily until these mice become hairless. The phenotypes are likely due to effects on expression patterns and levels due to transgene copy number and integration site differences (Table 1). Indeed, initial characterization of the three lines in brain tissue showed substantial differences in miR-183 cluster miRNA abundance by RT-PCR (Tg^1MDW^ >>> Tg^2MDW^ > Tg^3MDW^, Supplementary Figure 1A). Also, *in-situ* hybridization (ISH) with an LNA-DIG labeled probe against miR-182 was consistent with RT-PCR, showing Bergmann glial cell localization in Tg^1MDW^ cerebellum only, a common cell type expressing GFAP-core promoter transgenes^23^ (Supplementary Figure 1B–D).

### MicroRNA 183 cluster is misexpressed in Tg^1MDW/1MDW^ SCs

**Figure 1:**
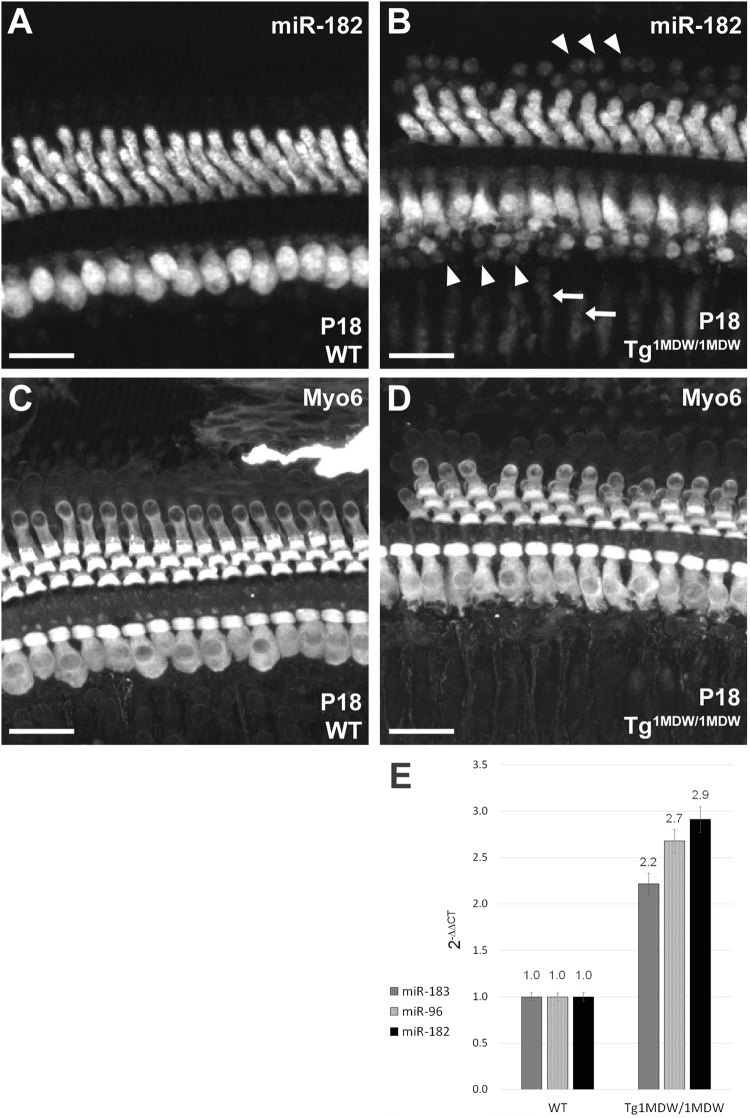
Supporting cell (SC) expression of miR-182 in Tg^1MDW/1MDW^ mice at P18. (**A**–**D**) Dual ISH/IHC fluorescence imaging of whole mount organ of Corti (OC) from WT (**A**,**C**) and Tg^1MDW/1MDW^ (**B**,**D**) P18 littermates. Cy5 fluorescence of tyramide enhanced miR-182 ISH labeling shows nuclear/cytoplasmic staining in hair cells (HCs **A**,**B**) and definitive Tg^1MDW/1MDW^ OC SC nuclear staining, including Deiters’, inner pillar, outer pillar, inner phalangeal and inner border cells (B, arrowheads). SC labeling with miR-182 was found in spiral limbus cells and myelinated Schwann cells of spiral ganglion neurons (**B**, arrows). Alexa 546 immunofluorescence of MYO6 positive HCs (**C**,**D**). (**E**) Quantitative increase in miR-183 cluster in P18 Tg^1MDW/1MDW^ total cochlear RNA by RT-PCR. Using ABI Taqman assays, quantitative RT-PCR was performed in Tg^1MDW/1MDW^ versus WT littermates (n = 3). The miR-183, miR-96 and miR-182 levels were normalized to Sno135. The results quantitate statistically significant (ΔCT values, 2 sample t-test, P < 0.001) increases in miR-182 (2.9 fold), miR-96 (2.7 fold) and miR-183 (2.2 fold) in Tg^1MDW/1MDW^ cochlea at P18. These data are consistent with a transgenic misexpression of these neurosensory miRNAs via the core human GFAP promoter and validate microarray data. Scale bar: 20 μM (**A**–**D**).

Loss of Preyer’s reflex in Tg^1MDW/1MDW^ adults led us to concentrate our inner ear characterizations to this genotype. We confirmed transgenic SC miR-183 cluster expression directly by dual whole mount ISH/IHC using LNA-DIG labeled probes against miR-182 and an antibody against MYO6 (Fig. 1A–D). Compared to the WT staining of miR-182 (Fig. 1A) that was restricted to inner and outer HCs (Fig. 1C), nuclear miR-182 labeling was found in all Tg^1MDW/1MDW^ SCs (Fig. 1B, arrowheads), including Deiters, inner pillar, outer pillar, inner phalangeal and inner border cells. Spiral limbus and Schwann cell nuclei also were stained with LNA-miR-182, similar to other GFAP promoter driven transgenes (Fig. 1B, arrows, Smeti *et al*., 2011). Unfortunately, we were unable to identify clear differences in miR-183 cluster expression in WT versus Tg^1MDW/1MDW^ at earlier ages (i.e. P5 and P10, data not shown). The Tg^1MDW^ construct lacked a separate reporter gene (e.g. LacZ, GFP) for this purpose. However, further evidence of transgenic SC miR-183 cluster expression was obtained through quantitative RT-PCR, which showed 2.9-, 2.7- and 2.2-fold higher levels of miR-182, miR-96 and miR-183, respectively, in Tg^1MDW/1MDW^ versus WT P18-cochlear total RNA (Fig. 1E). Taken together, these results confirm ectopic miR-183 cluster expression in OC SCs, spiral limbus cells and the Schwann cells that ensheathe neuronal processes projecting to and from the OC.

### Tg^1MDW/1MDW^ OC show increased cochlear IHCs and MYO6 positive Deiter’s Cells

**Figure 2:**
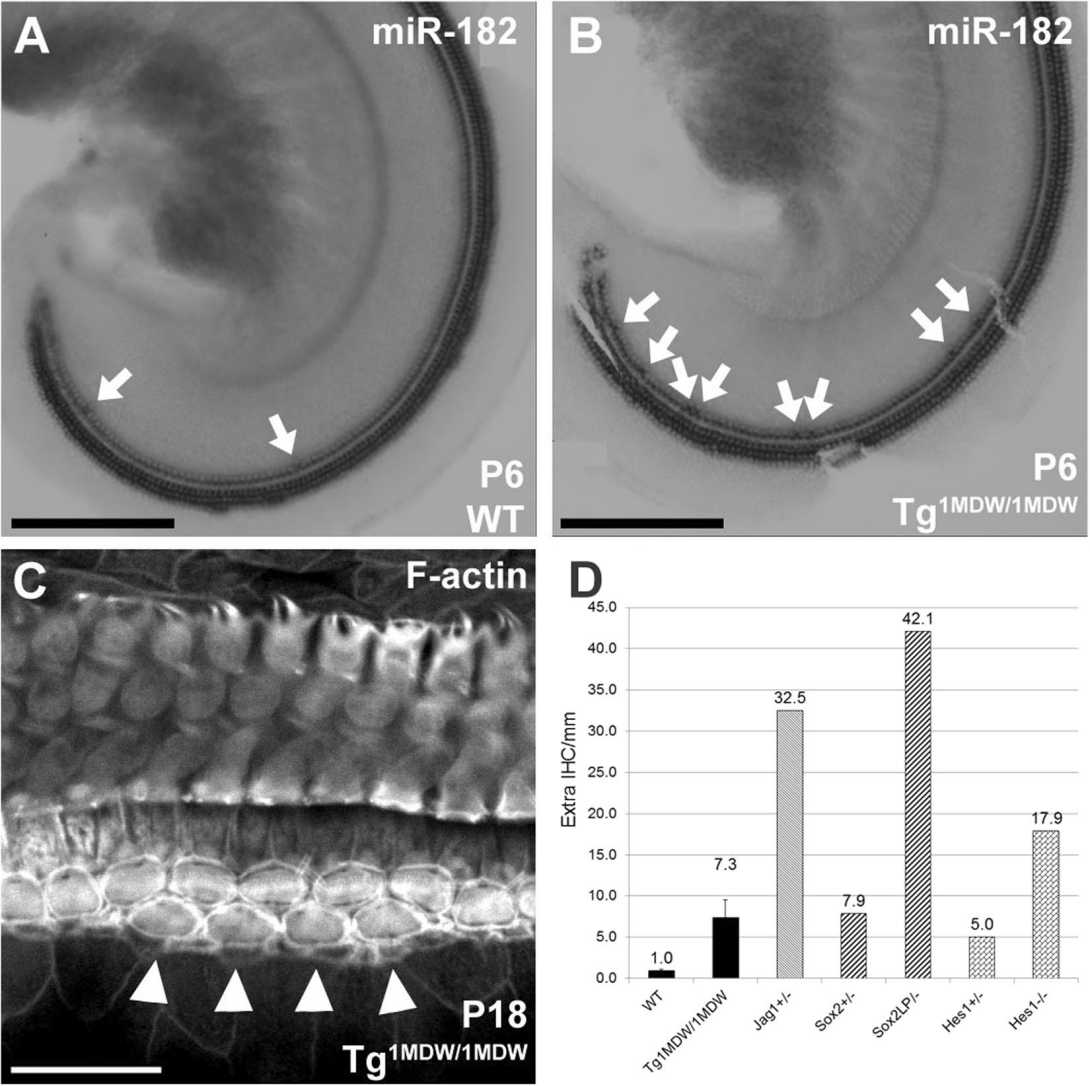
Excess IHCs are a consistent phenotype of prosensory/SC gene mutations. (**A**,**B**) Whole mount miR-182 staining of HCs in the cochlear apex of P6 WT (**A**) and Tg^1MDW/1MDW^ (**B**) mice. Arrows point to additional miR-182 positive, medially placed IHCs. (**C**) Phalloidin-stained Tg^1MDW/1MDW^ OC at P18 with 4 contiguous, medially placed extra IHCs (arrow heads). (**D**) Mean extra IHCs per 1 mm distance was plotted and compared with other published mouse mutants. Mutations in genes that are predicted targets of the miR-183 cluster phenocopy Tg^1MDW/1MDW^-mediated increases in IHCs. Phenotypic correlation of extra IHCs in SC-specific single-gene mutants are consistent with negative regulation of these predicted miR-183 cluster target genes. Scale bar: 200 μM (**A**,**B**); 20 μM (**C**).

**Figure 3:**
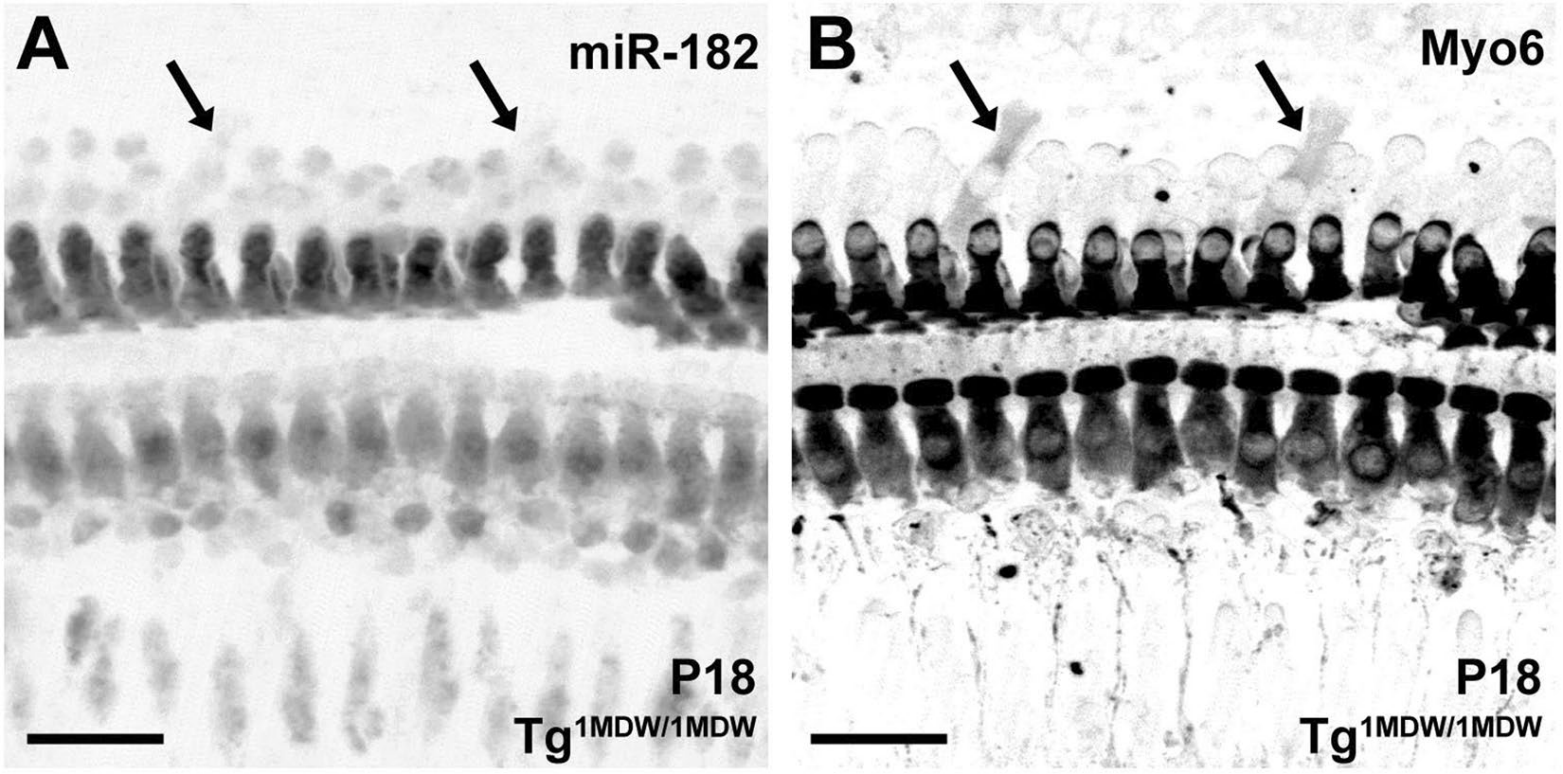
Deiter’s cell expression of MYO6 in P18 Tg^1MDW/1MDW^ OC, an early marker of differentiated HCs. (**A**) MYO6 staining in OC whole mount showing definitive label in Deiter’s cells (black arrowheads). (**B**) miR-182 co-staining. MYO6 is a marker of HC differentiation, suggesting that miR-183 cluster misexpression and its cytoplasmic localization are positive effectors of HC identity. Scale bar: 20 μM (**A**,**B**).

Even though we could not detect Tg^1MDW/1MDW^ misexpression at early postnatal ages by ISH, a clear histomorphological change in Tg^1MDW/1MDW^ OC were observed around P6 (Fig. 2A,B). A modest increase in apparent IHCs was found (Fig. 2B, arrows). Because cells in the OC undergo significant postmitotic functional maturation and programmed apoptosis occurs medially in the greater epithelial ridge, we chose P18 to quantify the expression of additional IHCs. When compared with WT littermates, Tg^1MDW/1MDW^ had an average of 7.2 extra IHC/mm of apical OC versus 0.97 extra IHC/mm in WT (Fig. 2C,D, n = 3 ears, T-test P = 0.038). The presence of additional HCs in this mouse model of SC miR-183 cluster misexpression is consistent with previous studies in both zebrafish and chicken where overexpression of miR-183 cluster miRNAs increased inner ear HC numbers^11,12^. Interestingly, this extra IHC patterning in Tg^1MDW/1MDW^ phenocopies both hypomorphic and null mutants of genes (i.e. *Jag1*, *Sox2*, *Hes1*) that are: 1) markers of differentiated SCs and; 2) predicted targets of miR-183 cluster miRNAs (Fig. 2D)^25–29^. For *Jag1*^+/−^, the extra IHCs estimate was extrapolated from counts of adult basal cochleae of headturner (*Jag1*^+*/Htu*^) and slalom (*Jag1*^+*/Slm*^) heterozygous mice^26,27^. For *Sox2*, extra IHCs were similarly extrapolated from counts taken from the entire length of late embryonic (E18) *Sox2*^*+/EGFP*^ reporter knockout (KO) and *Sox*^*EGFP/LP*^ hypomorphic cochleae^28^. For *Hes1*, counts were from 1 mm lengths starting from the basal end of E18 cochleae of KO *Hes1*^+/−^ and *Hes1*^*−/−*^ mice^25^. While the mechanism for the genesis of these extra IHCs is unknown, one possibility is that the transgenic miR-183 cluster expression promotes SC-to-HC transdifferentiation. In support of this idea, we detected a discrete number of apparent Deiters’ cells positive for both MYO6 and miR-182 at P18 (Fig. 3A,B, arrows). The cells appear to be histomorphologically hybrid in nature by: 1) showing decreased MYO6 staining compared to HCs; 2) having endfeet in contact with the basilar membrane, a characteristic of all OC SCs and; 3) displaying nuclei located at the same z-plane as OHC nuclei.

### miR-183 cluster mediated translational repression of select target genes

**Figure 4:**
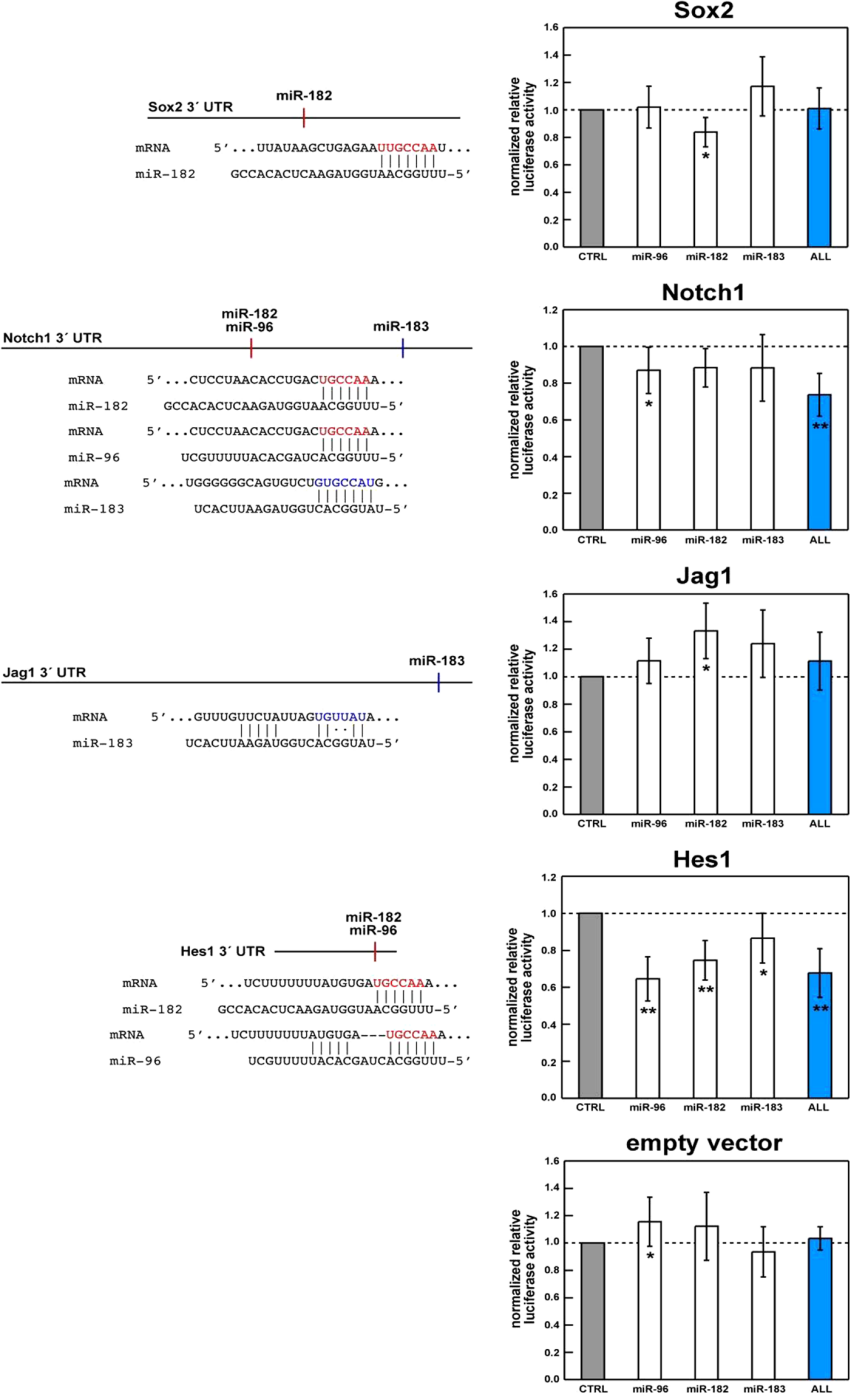
Validation of *Sox2*, *Notch1*, and *Hes1* as miR-183 cluster by dual luciferase assays. Histograms of mean relative luciferase activity in HEK293 cells co-transfected with a dual reporter vector (pmirGLO) containing cloned DNA sequences corresponding to the 3′ UTR of the indicated genes plus synthetic miRNA duplexes representing miR-96, miR-182, miR-183, or all three (ALL) normalized to scrambled siRNA control (CTRL). DNA sequences corresponding to the 3′ UTRs of *Sox2*, *Notch1*, *Jag1*, and *Hes1* were inserted downstream of the pmirGLO Photinus open reading frame. For each assay, the ratio of Photinus and Renilla luciferase activity in cells co-transfected with reporter vector and synthetic miRNA was normalized to that of cells transfected with reporter vector and scrambled siRNA. Each bar represents two replicate readings from each of six transfections performed over three experiments. Error bars indicate the standard error of the normalized mean. The Wilcoxon signed rank test was used to determine statistically significant differences in relative luciferase activity compared to control. Asterisks indicate P < 0.01 (*) and P < 0.005 (**).

To determine whether miR-183, miR-182, or miR-96 directly regulate select SC genes important to HC/SC differentiation (i.e. *Jag1*, *Sox2*, *Hes1, Notch1*) dual luciferase assays were performed. The respective 3′ UTRs were PCR amplified and cloned into the pmirGLO vector immediately downstream of the Photinus luciferase coding sequence. Each were co-transfected in HEK293 cultures with synthetic RNA duplexes representing miR-96, miR-182, or miR-183 alone or in combination. Scrambled miRNA duplexes were co-transfected with the reporter vector as a control. Luciferase activity for each experimental replicate was normalized to that of the scrambled control siRNA transfected replicate (CTRL) (Fig. 4). As expected, relative luciferase activity in cells co-transfected with miR-182 and reporter vector containing the *Sox2* 3′ UTR is reduced by nearly 20%, confirming previous findings that *Sox2* is a miR-183 cluster target^9^. Luciferase activity is significantly repressed in cells co-transfected with synthetic miR-96 or miR-183 and pmirGLO-Notch1 3′ UTR, suggesting that these miR-183 cluster members directly interact with and silence *Notch1*. Similarly, *Hes1* validates as a target of miR-96 and miR-182, with miR-96 downregulation of luciferase activity exceeding 20%. *Jag1*, however, failed to validate as a target of any miR-183 cluster member with statistical significance. This is likely due to G:U wobble base pairs at nucleotides 4 and 5 of miR-183 which, while thermodynamically favorable, can drastically reduce the efficacy of miRNA-mediated translational repression^30^. Taken together, these results suggest that *Sox2*, *Notch1*, and *Hes1* are genuine miR-183 cluster targets. Sox2 and Atoh1 exhibit a mutually antagonistic relationship in SC/HC differentiation^28^, while Notch1-regulated *Hes1* gene expression also antagonizes the ability of Atoh1 to promote hair cell differentiation^31^. Given that miR-183 cluster expression requires Atoh1-mediated HC specification^9^, the miR-183 cluster likely serves a crucial function in HC differentiation by downregulating *Sox2*, *Notch1*, and *Hes1*, thus contributing to Atoh1 specific HC fate determination.

### Microarray analysis reveals miR-183 cluster-specific changes in P5-OC transcriptome

**Figure 5:**
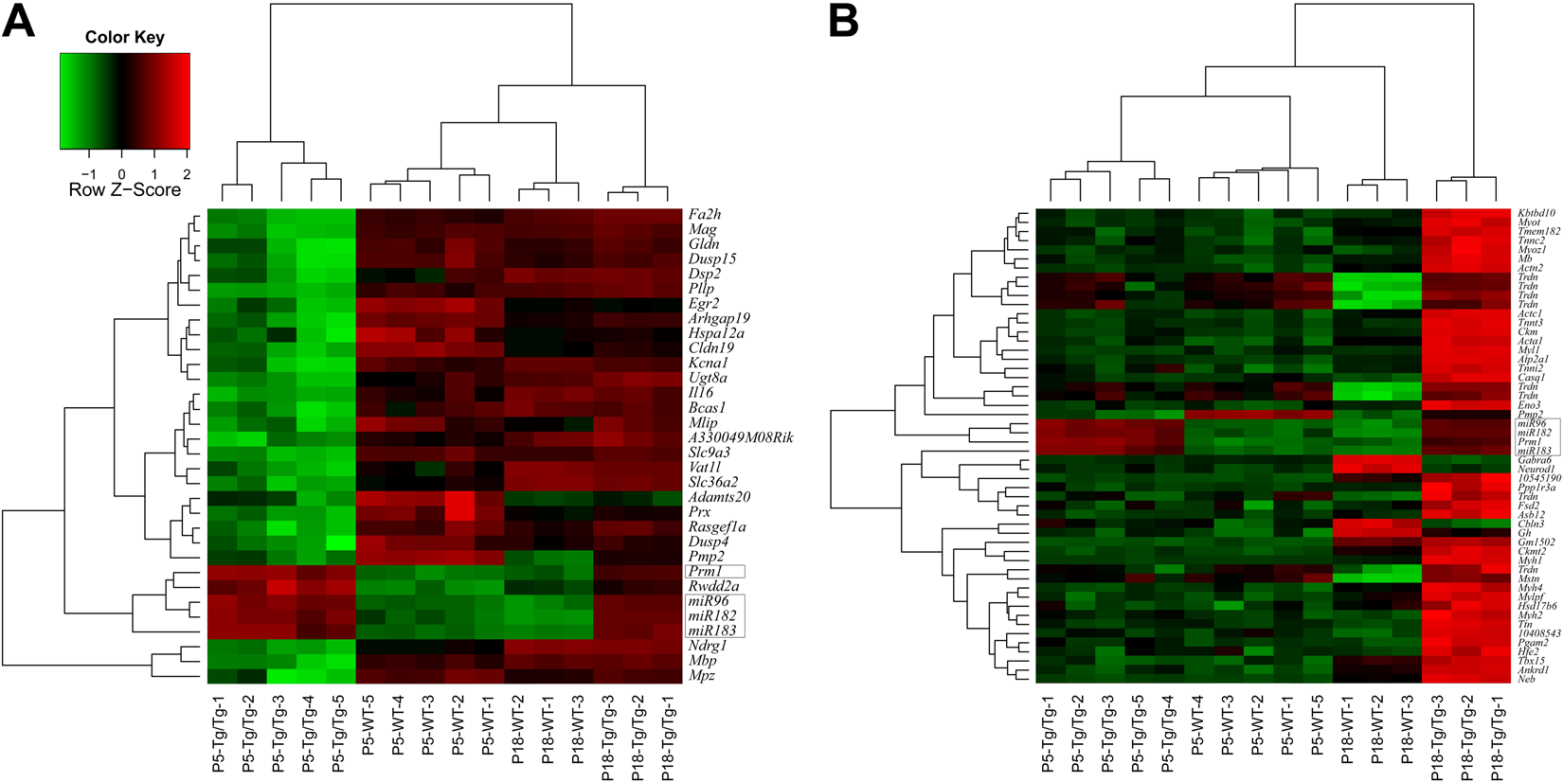
Clustered heatmaps of significantly changed genes in Tg^1MDW/1MDW^ Affymetrix microarray analysis. (**A**) OC-P5 heatmap showing normalized expression for 32 significantly changed genes found by two-way ANOVA (Age X Genotype, FDR-adjusted P < 0.05, fold change cutoff ± 2). (**B**) Cochlear-P18 heatmap of normalized expression for 52 probesets (45 genes) significantly changed in Tg^1MDW/1MDW^ (FDR-adjusted P < 0.05, fold change cutoff ±2). Irrespective of age, the most upregulated Affymetrix probesets were genetic elements of Tg^1MDW^: *Mir183*, *Mir96*, *Mir182* and *Prm1* (boxed probesets).

The mutual exclusion hypothesis of miRNA function^16^ predicts that miR-183 cluster misexpression in adjacent and lineage-related SCs should repress evolutionarily conserved target genes essential to SC identity/function. To test for this type of aggregate effect, we performed whole-genome transcription profiling using Affymetrix microarrays (MoGene 1.0 microarrays) to evaluate mRNA expression in Tg^1MDW/1MDW^ versus WT in the P5-OC (n = 5) and P18-cochlea (n = 3). Two-way ANOVA testing found significant differences (FDR-adjusted P < 0.05, fold change cutoff ± 2) in Tg^1MDW/1MDW^ versus WT littermates at either P5 (Table 2, Fig. 5A) or P18 (Table 3, Fig. 5B). Irrespective of age, the most upregulated Affymetrix probesets were genetic elements of Tg^1MDW^: *Mir183*, *Mir96*, *Mir182* and *Prm1* (Fig. 5A,B, boxed probesets), with fold changes + 5.67 (P18-*Prm1*) to + 16.97 (P18-*Mir182*). In the P5-OC, the majority of changed genes (27/32) were downregulated (Fig. 5A, Table 2), whereas in P18-cochlea, the majority were upregulated (37/45), not counting *Prm1* and miR-183 cluster miRNAs (Fig. 5B, Table 3).

**Table 2:** List of 32 significantly changed Tg^1MDW/1MDW^ Affymetix probesets in the P5-OC. From a total of 28,188 annotated probesets using the Limma Package and MicroArraysRUS Gui interface to R and Bioconductor microarray analysis programs.

AffyID	GeneID	GeneName	EntrezID	Tg^1MDW/1MDW^ to WT Ratio	FDR p-value
10437684	Prm1	protamine 1	19118	12.79	4.7E-08
10543680	Mir96	microRNA 96	723886	11.00	4.1E-07
10543682	Mir182	microRNA 182	387177	10.57	4.7E-08
10543684	Mir183	microRNA 183	387178	9.91	6.8E-07
10587604	Rwdd2a	RWD domain containing 2 A	69519	5.44	8.7E-07
10457022	Mbp	myelin basic protein	17196	−2.04	4.9E-05
10490061	Bcas1	breast carcinoma amplified sequence 1	76960	−2.07	1.2E-03
10468746	Hspa12a	heat shock protein 12 A	73442	−2.07	1.9E-03
10601729	Drp2	dystrophin related protein 2	13497	−2.12	1.1E-02
10517364	A330049M08Rik	RIKEN cDNA A330049M08 gene	230822	−2.12	2.3E-04
10575693	Vat1l	vesicle amine transport protein 1 homolog-like (T. californica)	270097	−2.15	1.5E-03
10565218	Il16	interleukin 16	16170	−2.15	3.7E-06
10429140	Ndrg1	N-myc downstream regulated gene 1	17988	−2.24	3.5E-05
10595094	2310046A06Rik	RIKEN cDNA 2310046A06 gene	69642	−2.35	3.4E-04
10351525	Mpz	myelin protein zero	17528	−2.37	1.9E-03
10571312	Dusp4	dual specificity phosphatase 4	319520	−2.50	4.0E-04
10541114	Rasgef1a	RasGEF domain family, member 1 A	70727	−2.59	3.5E-05
10551365	Prx	periaxin	19153	−2.69	1.1E-05
10431749	Adamts20	a disintegrin-like and metallopeptidase (reprolysin type) with thrombospondin type 1 motif, 20	223838	−2.78	4.0E-04
10501963	Ugt8a	UDP galactosyltransferase 8 A	22239	−2.84	5.7E-05
10363735	Egr2	early growth response 2	13654	−3.01	3.0E-05
10386020	Slc36a2	solute carrier family 36 (proton/amino acid symporter), member 2	246049	−3.19	6.7E-05
10467637	Arhgap19	Rho GTPase activating protein 19	71085	−3.23	3.1E-05
10580765	Pllp	plasma membrane proteolipid	67801	−3.31	6.1E-08
10548047	Kcna1	potassium voltage-gated channel, shaker-related subfamily, member 1	16485	−3.35	5.4E-04
10585398	Gldn	gliomedin	235379	−3.52	8.6E-04
10488678	Dusp15	dual specificity phosphatase-like 15	252864	−3.68	8.0E-05
10406176	Slc9a3	solute carrier family 9 (sodium/hydrogen exchanger), member 3	105243	−3.84	4.1E-07
10581824	Fa2h	fatty acid 2-hydroxylase	338521	−3.93	1.4E-06
10507635	Cldn19	claudin 19	242653	−4.09	2.3E-06
10562152	Mag	myelin-associated glycoprotein	17136	−4.70	4.1E-07
10497253	Pmp2	peripheral myelin protein 2	18857	−7.49	2.8E-06

**Table 3:** List of 52 significantly changed Tg^1MDW/1MDW^ changed Affymetix probesets in the P18-cochlea. From a total of 28,188 annotated probesets using the Limma Package and MicroArraysRUS Gui interface to R and Bioconductor microarray analysis programs.

AffyID	GeneID	GeneName	EntrezID	Tg^1MDW/1MDW^ to WT Ratio	FDR p-value
10543680	Mir96	microRNA 96	723886	16.97	2.7E-07
10567879	Atp2a1	ATPase, Ca + + transporting, cardiac muscle, fast twitch 1	11937	12.29	2.3E-08
10543684	Mir183	microRNA 183	387178	11.10	1.6E-06
10377087	Myh1	myosin, heavy polypeptide 1, skeletal muscle, adult	17879	10.86	1.7E-11
10355259	Myl1	myosin, light polypeptide 1	17901	10.46	2.6E-10
10543682	Mir182	microRNA 182	387177	10.28	1.9E-07
10362422	Trdn	triadin	76757	7.69	3.8E-06
10559221	Tnnt3	troponin T3, skeletal, fast	21957	7.55	2.8E-08
10485982	Actc1	actin, alpha, cardiac muscle 1	11464	5.68	7.5E-07
10437684	Prm1	protamine 1	19118	5.67	6.4E-06
10417933	Myoz1	myozenin 1	59011	5.65	8.9E-07
10360235	Casq1	calsequestrin 1	12372	5.30	2.7E-07
10362434	Trdn	triadin	76757	5.24	3.3E-05
10362428	Trdn	triadin	76757	5.04	6.6E-04
10550698	Ckm	creatine kinase, muscle	12715	4.98	1.6E-07
10430140	Mb	myoglobin	17189	4.30	2.7E-07
10362420	Trdn	triadin	76757	4.25	2.1E-06
10408543	NA	NA	NA	4.19	2.4E-06
10377938	Eno3	enolase 3, beta muscle	13808	3.86	1.4E-05
10362442	Trdn	triadin	76757	3.80	4.8E-04
10362418	Trdn	triadin	76757	3.80	5.5E-04
10582592	Acta1	actin, alpha 1, skeletal muscle	11459	3.79	4.3E-05
10410984	Ckmt2	creatine kinase, mitochondrial 2	76722	3.69	7.0E-07
10489545	Tnnc2	troponin C2, fast	21925	3.57	4.3E-05
10472562	Kbtbd10	kelch repeat and BTB (POZ) domain containing 10	228003	3.54	1.7E-06
10545190	NA	NA	NA	3.52	6.4E-04
10467191	Ankrd1	ankyrin repeat domain 1 (cardiac muscle)	107765	3.49	3.3E-05
10482528	Neb	nebulin	17996	3.44	9.8E-09
10362404	Trdn	triadin	76757	3.39	3.3E-05
10455461	Myot	myotilin	58916	3.34	1.1E-06
10362436	Trdn	triadin	76757	3.18	1.7E-03
10377055	Myh2	myosin, heavy polypeptide 2, skeletal muscle, adult	17882	3.04	3.8E-06
10407742	Actn2	actinin alpha 2	11472	2.99	3.0E-06
10497253	Pmp2	peripheral myelin protein 2	18857	2.98	8.0E-03
10345869	Tmem182	transmembrane protein 182	381339	2.91	8.1E-05
10377117	Myh4	myosin, heavy polypeptide 4, skeletal muscle	17884	2.89	1.4E-05
10483871	Ttn	titin	22138	2.88	2.7E-07
10543233	Ppp1r3a	protein phosphatase 1, regulatory (inhibitor) subunit 3 A	140491	2.86	1.6E-05
10384015	Pgam2	phosphoglycerate mutase 2	56012	2.75	1.6E-06
10559200	Tnni2	troponin I, skeletal, fast 2	21953	2.48	2.2E-04
10346250	Mstn	myostatin	17700	2.39	1.6E-05
10373334	Hsd17b6	hydroxysteroid (17-beta) dehydrogenase 6	27400	2.38	9.0E-03
10557575	Mylpf	myosin light chain, phosphorylatable, fast skeletal muscle	17907	2.37	1.1E-04
10494672	Tbx15	T-box 15	21384	2.26	2.6E-03
10605815	Asb12	ankyrin repeat and SOCS box-containing 12	70392	2.21	1.3E-05
10565137	Fsd2	fibronectin type III and SPRY domain containing 2	244091	2.16	9.3E-03
10545187	Gm1502	predicted gene 1502	385120	2.03	1.2E-04
10494423	Hfe2	hemochromatosis type 2 (juvenile) (human homolog)	69585	2.03	6.4E-04
10420209	Cbln3	cerebellin 3 precursor protein	56410	−2.00	1.1E-04
10392135	Gh	growth hormone	14599	−2.45	3.4E-03
10484276	Neurod1	neurogenic differentiation 1	18012	−3.53	5.0E-07
10385310	Gabra6	gamma-aminobutyric acid (GABA) A receptor, subunit alpha 6	14399	−3.64	1.1E-09

To provide a biologic context to these expression changes, we performed gene ontology (GO) analysis using the DAVID bioinformatics resources^32^. For this, we submitted gene lists that included changed genes down to a ± 1.5-fold change cutoff (FDR-adjusted P < 0.05). Pathway analysis revealed P5 downregulated genes were significantly enriched for genes involved in myelination and ion homeostasis; P18 downregulated genes were related to synapse structure; and P18 upregulated genes were related to muscle and the actin cytoskeleton (Table 4). To evaluate transcriptome effects directly attributable to the miR-183 cluster, we performed hypergeometric analyses using a non-redundant set of evolutionarily conserved miRNA/3′UTR seed sequences: 153 vertebrate miRNAs and 62,793 3′UTR seed sites (8mer, 7mer-m8, 7mer-1A, 3comp, TargetScan 6.2^29^). This seed site universe was used to test for enrichment/depletion within upregulated and downregulated gene sets. For each miR-183 cluster miRNA, we found enrichment (Odds Ratio 1.43–1.45, FDR-adjusted P < 0.05, Table 5) of evolutionarily conserved 3′UTR seed targets in P5 Tg^1MDW/1MDW^ downregulated genes (uncorrected P < 0.05, fold change <−1.05). Importantly, this enrichment is consistent with repression of miR-183 cluster target genes at evolutionarily conserved sites in Tg^1MDW/1MDW^ P5-OC. In P18-cochlea, seed sites of 23 and 6 vertebrate miRNAs were either enriched (Odd Ratio >1, FDR-adjusted P < 0.05) or depleted (Odds Ratio <1, FDR-adjusted P < 0.05) in P18-cochlea upregulated or downregulated gene sets, respectively (uncorrected P < 0.05, fold change <−1.05 or >+ 1.05, Table 5). Only miR-96 seed sites were found depleted in upregulated genes at P18, an effect opposite to that observed for this miRNA at P5-OC.

**Table 4:** Overrepresented Gene Ontology (G) terms for upregulated and downregulated genes in P5-OC and P18-cochlea. FDR < 0.05, fold-change ± 1.5. Gene totals (Up|Down): P5-OC (27|93); P18-cochlea (15|86).

Age	Direction	GO Term	Fold Enrichment	Genes	P-Value (Bonferroni)
P5	Down	myelin sheath	92.90	*Mag,Mbp,Plp1,Gjc3*	9.1E-04
		myelination	50.92	*Pmp22,Fyn,Mbp,Ugt8a,Egr2,Plp1,Lgi4,Gjc3,Pou3f1*	2.4E-09
		ensheathment of neurons	48.31	*Pmp22,Fyn,Mbp,Ugt8a,Egr2,Plp1,Lgi4,Gjc3,Pou3f1*	3.8E-09
		axon ensheathment	48.31	*Pmp22,Fyn,Mbp,Ugt8a,Egr2,Plp1,Lgi4,Gjc3,Pou3f1*	3.8E-09
		regulation of action potential in neuron	41.87	*Pmp22,Fyn,Mbp,Ugt8a,Egr2,Plp1,Lgi4,Gjc3,Pou3f1*	1.3E-08
		regulation of action potential	34.89	*Pmp22,Fyn,Mbp,Ugt8a,Egr2,Plp1,Lgi4,Gjc3,Pou3f1*	6.1E-08
		regulation of membrane potential	17.59	*Pmp22,Fyn,Mbp,Ugt8a,Egr2,Plp1,Lgi4,Gjc3,Shtn1,Pou3f1*	1.8E-06
		transmission of nerve impulse	12.15	*Sv2c,Lgi4,Plp1,Gjc3,Cldn19,Pou3f1,Fyn,Syn3,Pmp22,Fnta,Mbp,Ugt8a,Egr2*	2.0E-07
		cellular ion homeostasis	8.18	*Pmp22,Fyn,Mbp,Ugt8a,Egr2,Plp1,Lgi4,Gjc3,Shtn1,Pou3f1*	1.3E-03
		ion homeostasis	8.00	*Pmp22,Fyn,Slc9a3,Mbp,Ugt8a,Egr2,Plp1,Lgi4,Gjc3,Shtn1,Pou3f1*	3.9E-04
		cellular chemical homeostasis	7.96	*Pmp22,Fyn,Mbp,Ugt8a,Egr2,Plp1,Lgi4,Gjc3,Shtn1,Pou3f1*	1.6E-03
		chemical homeostasis	6.40	*Pmp22,Fyn,Slc9a3,Mbp,Ugt8a,Egr2,Plp1,Lgi4,Gjc3,Shtn1,Pou3f1*	2.8E-03
		cellular homeostasis	6.19	*Pmp22,Fyn,Mbp,Ugt8a,Egr2,Plp1,Lgi4,Gjc3,Shtn1,Pou3f1*	1.2E-02
P5	Up	None			
P18	Down	synapse	17.09	*Gria1,Gabra6,Cbln1,Cbln3*	3.6E-02
P18	Up	striated muscle thin filament	145.82	*Actn2,Neb,Tpm2,Acta1*	2.7E-04
		A band	85.06	*Ttn,Myh2,Myom1,Atp2a1,Myh1*	1.7E-03
		myofibril assembly	68.11	*Actc1,Ttn,Neb,Acta1*	8.7E-03
		actomyosin structure organization	51.76	*Actc1,Ttn,Neb,Acta1*	2.0E-02
		sarcomere	41.47	*Actc1,Myh2,Ttn,Neb,Myot,Myom1,Atp2a1,Myh4,Myoz2,Myh1,Actn2,Tpm2,Acta1,Ankrd1*	1.6E-14
		myofibril	39.26	*Actc1,Myh2,Ttn,Neb,Myot,Myom1,Atp2a1,Myoz2,Myh4,Myh1,Nrap,Actn2,Tpm2,Acta1,Ankrd1*	1.5E-15
		contractile fiber part	38.57	*Actc1,Myh2,Ttn,Neb,Myot,Myom1,Atp2a1,Myh4,Myoz2,Myh1,Actn2,Tpm2,Acta1,Ankrd1*	4.9E-14
		muscle contraction	37.74	*Ttn,Myh2,Actn2,Cacna1s,Myom1,Tpm2,Myh4,Myh1*	8.3E-06
		contractile fiber	37.61	*Actc1,Myh2,Ttn,Neb,Myot,Myom1,Atp2a1,Myoz2,Myh4,Myh1,Nrap,Actn2,Tpm2,Acta1,Ankrd1*	2.7E-15
		sarcoplasmic reticulum	37.53	*Trdn,Srl,Cacna1s,Atp2a1,Casq1*	1.2E-03
		sarcoplasm	35.44	*Trdn,Srl,Cacna1s,Atp2a1,Casq1*	1.5E-03
		muscle system process	33.80	*Ttn,Myh2,Actn2,Cacna1s,Myom1,Tpm2,Myh4,Myh1*	1.6E-05
		I band	33.70	*Actc1,Ttn,Actn2,Myot,Atp2a1,Myoz2,Ankrd1*	6.8E-06
		striated muscle cell development	29.96	*Actc1,Ttn,Neb,Cacna1s,Acta1*	7.3E-03
		muscle cell development	26.52	*Actc1,Ttn,Neb,Cacna1s,Acta1*	1.2E-02
		myosin complex	25.10	*Ttn,Myh2,Mylpf,Myom1,Myl1,Myh4,Myh1*	5.0E-04
		striated muscle tissue development	15.28	*Actc1,Ttn,Cacna1s,Mylpf,Acta1,Myf6*	1.4E-02
		actin cytoskeleton	14.94	*Myh2,Ttn,Neb,Mylpf,Myom1,Myh4,Myoz2,Myl1,Myh1,Actn2,Tpm2,Acta1,Myoz1*	3.4E-08
		muscle tissue development	14.27	*Actc1,Ttn,Cacna1s,Mylpf,Acta1,Myf6*	1.9E-02
		actin binding	7.85	*Myh2,Actn2,Tmod4,Myot,Tpm2,Myh4,Tnni2,Myh1,Nrap*	8.5E-03
		calcium ion binding	4.04	*Ttn,Cdh19,Actn2,Srl,Cacna1s,Mylpf,Plscr4,Atp2a1,Myl1,Casq1,Tnnc2,Prrg4*	1.6E-02
		cytoskeleton	3.64	*Actc1,Myh2,Ttn,Neb,Mylpf,Myot,Myom1,Myoz2,Myh4,Myl1,Myh1,Sgcg,Actn2,Tmod4,Tpm2,Acta1,Myoz1*	1.7E-03

**Table 5:** Hypergeometric analysis of genome-wide microarray data demonstrate miR-183 cluster specific enrichment/depletion of evolutionary miRNA/3′UTR seed targets.

Microarray	Mutant < WT. target enrichment	Odds Ratio	P-Value (FDR)	Mutant > WT exp. miRNA- enrichment	Odds Ratio	P-Value (FDR)
Tg^1MDW/1MDW^	miR-96	1.45	2.20E-06	miR-33	2.66	0.016
Tg 183 cluster (n = 5)	miR-182	1.43	4.70E-06	miR-376	3.48	0.016
P5-OC	miR-328	0.47	0.0065	miR-124	0.55	0.039
	miR-183	1.45	0.031			
Tg^1MDW/1MDW^	miR-326	2.32	0.011	miR-24	0.41	1.10E-06
Tg 183 cluster (n = 3)	miR-34	1.91	0.008	miR-326	0.38	1.90E-04
P18-cochlea	miR-200/429	0.49	0.010	miR-214	0.57	8.10E-04
	miR-129	1.98	0.011	miR-149	0.46	6.90E-04
	miR-210	6.67	0.017	miR-328	0.30	0.0016
	miR-328	2.40	0.041	miR-101	1.45	0.0019
				miR-340	1.32	0.0019
				miR-125	0.65	0.0020
				miR-22	0.54	0.0027
				miR-433	1.76	0.0043
				miR-491	0.29	0.0043
				miR-185	0.44	0.0050
				miR-653	2.03	0.0053
				miR-186	1.41	0.0060
				miR-34	0.64	0.0064
				miR-874	0.44	0.0065
				miR-485	0.50	0.0065
				miR-875	2.23	0.0111
				miR-29	0.75	0.0106
				miR-205	1.51	0.0140
				miR-200/429	1.27	0.0211
				miR-96	0.77	0.0251
				mir-217	1.50	0.0471
*miR-96*^*ddl/ddl*^ MUT miR-96 (n = 3)	none	N/A	N/A	miR-96	2.10	1.80E-06
P4 OC				miR-129	2.24	0.0030
*miR-183C* ^*GT/GT*^	miR-96	0.52	3.10E-04	miR-182	1.70	6.80E-08
KO183 (n = 3)	miR-182	0.58	0.0023	miR-96	1.67	1.60E-07
5wk retina	miR-433	2.04	0.036	miR-15	0.64	0.00046
				miR-543	1.51	0.012
				miR-33	1.70	0.013
				miR-183	1.65	0.013
				miR-125	0.66	0.013
				miR-208	1.98	0.017
				miR-122	0.32	0.033
				miR-23	0.72	0.033
				miR-103	0.64	0.042
				let-7	0.75	0.041

For validation of our methodology, we applied the same hypergeometric analysis of the TargetScan 6.2 seed site universe to published microarray data from P4-OC miR-96 Diminuendo mouse (*miR96*^*ddl/ddl*^)^2^ and from 5wk-retina miR-183 cluster KO mouse (*miR-183C*^*GT/GT*^)^33^. In the case of the *miR96*^*ddl/ddl*^ microarray, miR-96 seed sites were enriched (Odds Ratio 2.10, FDR-adjusted P < 0.05) in *miR96*^*ddl/ddl*^ upregulated genes (uncorrected P < 0.05, fold change >+ 1.05). This independently confirms specific de-repression of evolutionarily conserved miR-96 target genes due to a miR-96 single nucleotide mutation^2^. In *miR-183C*^*GT/GT*^ 5wk-retina, our hypergeometric analysis demonstrates that combined loss of all miR-183 cluster miRNAs results not only in enrichment (Odds Ratio 1.65–1.70, FDR-adjusted P < 0.05, Table 5) of miR-183 cluster targets in *miR-183C*^*GT/GT*^ upregulated genes (uncorrected P < 0.05, fold change >+ 1.05), but also the reciprocal depletion of miR-96 and miR-182 targets (Odds Ratio 0.52, 0.58, respectively, FDR-adjusted P < 0.05, Table 5) in *miR-183C*^*GT/GT*^ downregulated genes (uncorrected P < 0.05, fold change <−1.05). Taken together, the hypergeometric analysis of Tg^1MDW/1MDW^ demonstrates that at P5 in the OC, miR-183 cluster target genes, defined by both miRNA and 3′UTR seed site evolutionary conservation, are reduced concomitantly with transgenic overexpression of miR-183, mir-96 and miR-182.

**Table 6:** Tg-(GFAP-*Mir183,Mir96,Mir182*) genotyping primers and probes.

Name	Use	Sequence (5′-3′)	Length
Atoh1-U	PCR	CTGAAAACTGAGACAACCAAATGC	23
Atoh1-L	PCR	AAGGGTGCAGGGATATTTGTCA	21
Atoh1-Hex	5′ nuclease probe	HEX™-TCCTAGCGCGCGGGAAGCC-BHQ-1®	19
Tg-U	PCR	AACAGCCAGATCACCTTTCACTGC	24
Tg-L	PCR	GCGCTCTTCCCACAGTTAACACAA	24
Tg-Fam	5′ nuclease probe	6-FAM™-AGGGATATCGGGCTTGAGGAGGTTT-BHQ-1®	25

**Figure 6:**
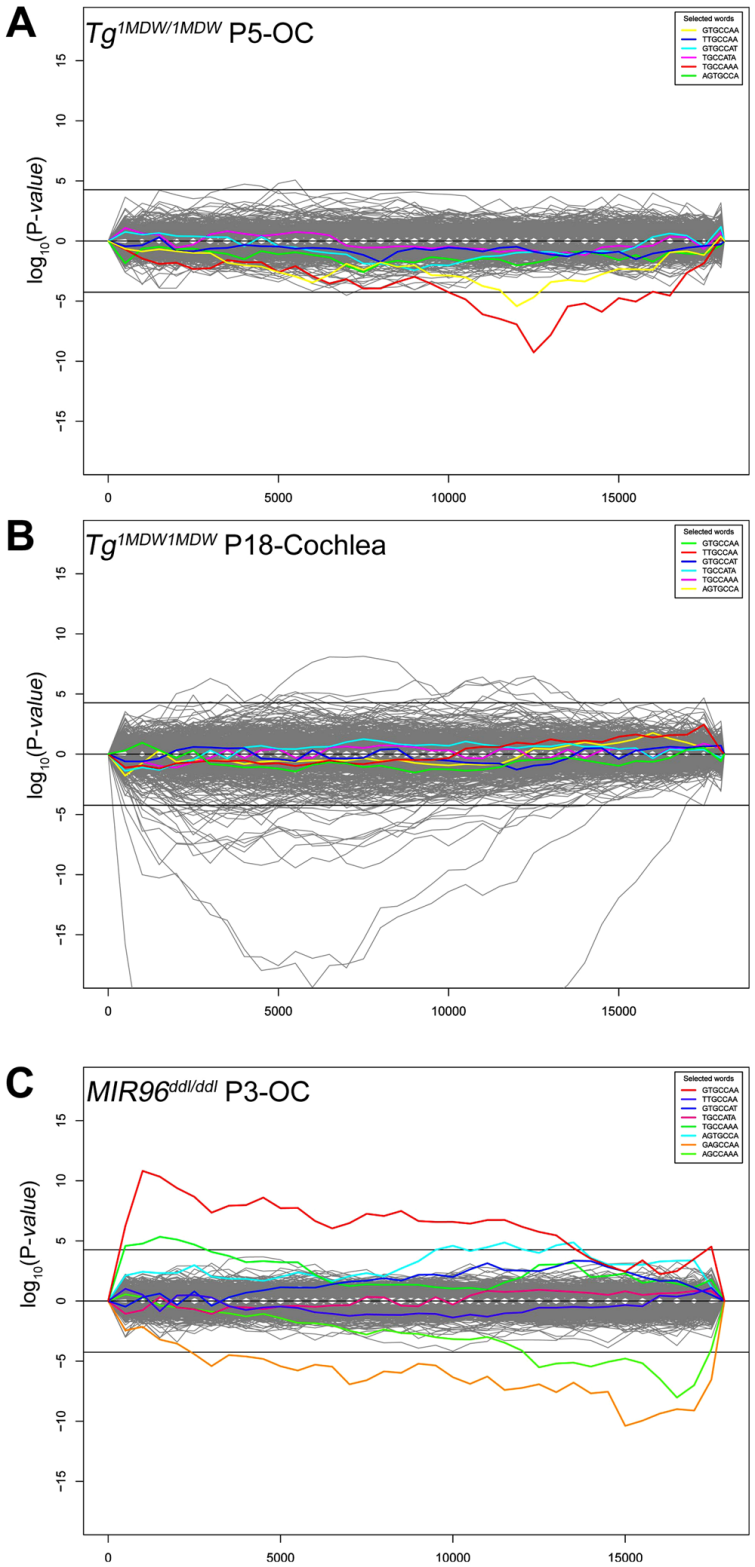
Sylamer analysis of microarray data showing enrichment and depletion for 911 miRNA heptamer seed sequences in 3′UTRs. (**A**) Tg^1MDW/1MDW^ P5-OC demonstrates significant depletion of 2 miR-183 cluster heptamers (GTGCCAA, TGCCAAA), validating this miRNA misexpression model. The x axis is the number of all microarray genes sorted from most upregulated (left) to most downregulated (right). The y axis shows the hypergeometric significance for enrichment or depletion of heptamers in 3′UTRs at leading parts of the gene list. Positive values indicate enrichment [-log_10_(P)] and negative values depletion [log_10_(P)]. Horizontal lines represent 0.05 Bonferroni P-value threshold. (**B**) Tg^1MDW/1MDW^ P18-cochlea microarray fails to demonstrate miR-183 cluster effects, but does show significant imbalances in 3′UTR heptamer representation for dozens of miRNA complementary sequences. (**C**) Analysis of available primary *Mir96*^*ddl/ddl*^ P3-OC microarray data (n = 3) confirms enrichment of miR-96^wt^ (GTGCCAA, TGCCAAA) and depletion of miR-96^ddl^ heptamers^2^. All highlighted plots are miR-183 cluster heptamers and include two (2) predicted to complement *Mir96*^*ddl*^ mutant miR-96. Sylamer program reference^34^.

While there appears to be compelling evidence for wider effects on conserved miRNA targets in P18-cochlea, the hypergeometric analysis did not reveal convincing miR-183 cluster effects in the P18-cochlea microarray data. One possibility is that miR-183 cluster primary effects on gene expression observed at P5 are masked by increasing secondary effects at P18. Alternatively, the miRNA/mRNA interactions could include non-conserved and/or off-target effects of the miR-183 cluster. To address the latter two possibilities, we employed the Sylamer program to identify, through hypergeometric analysis, over- or under-represented 7-mer sequence strings (seeds) in the mouse 3′UTR transcriptome interrogated by the Affymetrix MoGene 1.0 platform^34^. Sylamer differs from the previous Targetscan-based methodology in two important ways. The first is that all gene expression data is included, regardless of probeset P-values, to generate ranked gene-lists from most upregulated to most downregulated. The second is that evolutionary conservation of predicted miRNA seed sequences across homologous 3′UTRs is not a component in the analysis. Sylamer analysis of 912 miRNA target heptamers, including all conserved 8mer, 7mer-m8, 7mer-1A sites from TargetScan 6.2, revealed significant depletion of two miR-183 cluster target heptamers (GTGCCAA, TGCCAAA) in downregulated Tg^1MDW/1MDW^ P5-OC 3′UTRs (Fig. 6A), complementing the TargetScan 6.2-based hypergeometric analysis (Table 5, Fisher’s Exact Test P = 0.017). This transcriptome level microarray analysis validates the engineered intent of Tg^1MDW^, i.e. miR-183 cluster SC misexpression to downregulate genes that are disproportionate with respect to miR-183 cluster 3′UTR-bearing seed sequences. For P18-cochlea, Sylamer analysis showed no significant (conserved, non-conserved and/or off-target) effects attributable to miR-183 cluster (Fig. 6B). Nevertheless, 49 conserved miRNA target heptamers were enriched (17) or depleted (32) (Fig. 6B). However, these miRNA heptamer seeds were distinct from those conserved miRNAs identified by TargetScan 6.2-based hypergeometric analysis (Table 5, Fisher’s Exact Test P = 0.0033). As confirmation of our in-house application of Sylamer, we also analyzed the publicly available microarray data (n = 3), confirming enrichment of miR-96^wt^ heptamers (GTGCCAA, TGCCAAA) and depletion of miR-96^ddl^ heptamers (GAGCCAA, AGCCAAA) in upregulated and downregulated genes, respectively, in the diminuendo (*Dmdo* or *Mir96*^*ddl/ddl*^) OC at P4^2^ (Fig. 6C) in agreement with the TargetScan 6.2 based hypergeometric analysis (Table 5, Fisher’s Exact Test P = 0.013).

### Trangenic miRNA misexpression results in profound cochlear dysfunction

**Figure 7:**
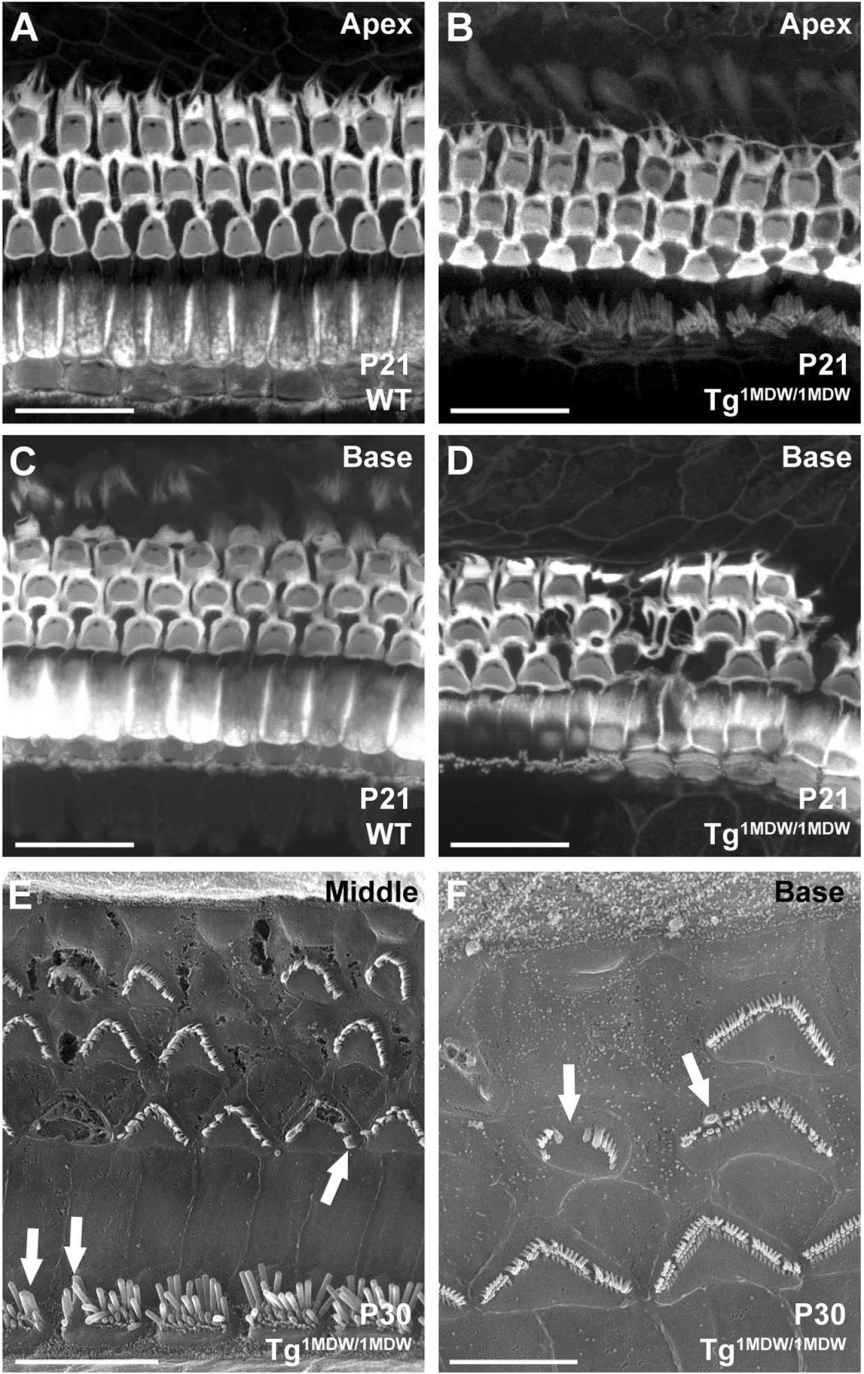
Tg^1MDW/1MDW^ OHC loss and stereocilia defects. (**A**–**D**) Confocal microscopy of MTRITC-Phalloidin-stained P21 WT and Tg^1MDW/1MDW^ cochlear tissue whole mounts. The images are brightest point projections of 7.6 micron Z-plane images parallel to the reticular lamina. (**A**,**B**) Apical OC shows comparable OHC/SC F-actin cytoarchitecture in WT versus Tg^1MDW/1MDW^ at P21. (**C**,**D**) In the basal OC, F-actin cytoarchitecture is disturbed due to missing OHCs in Tg^1MDW/1MDW^ at P21. (**E**,**F**) SEM images of Tg^1MDW/1MDW^ OC reticular lamina at P30 showing stereocilia defects in both OHC and IHCs, including absence of stereocilia and stereocilia fusion (arrows). Scale bar; 20 μM (**A**–**D**), 10 μM (**E**), 5 μM (**F**).

**Figure 8:**
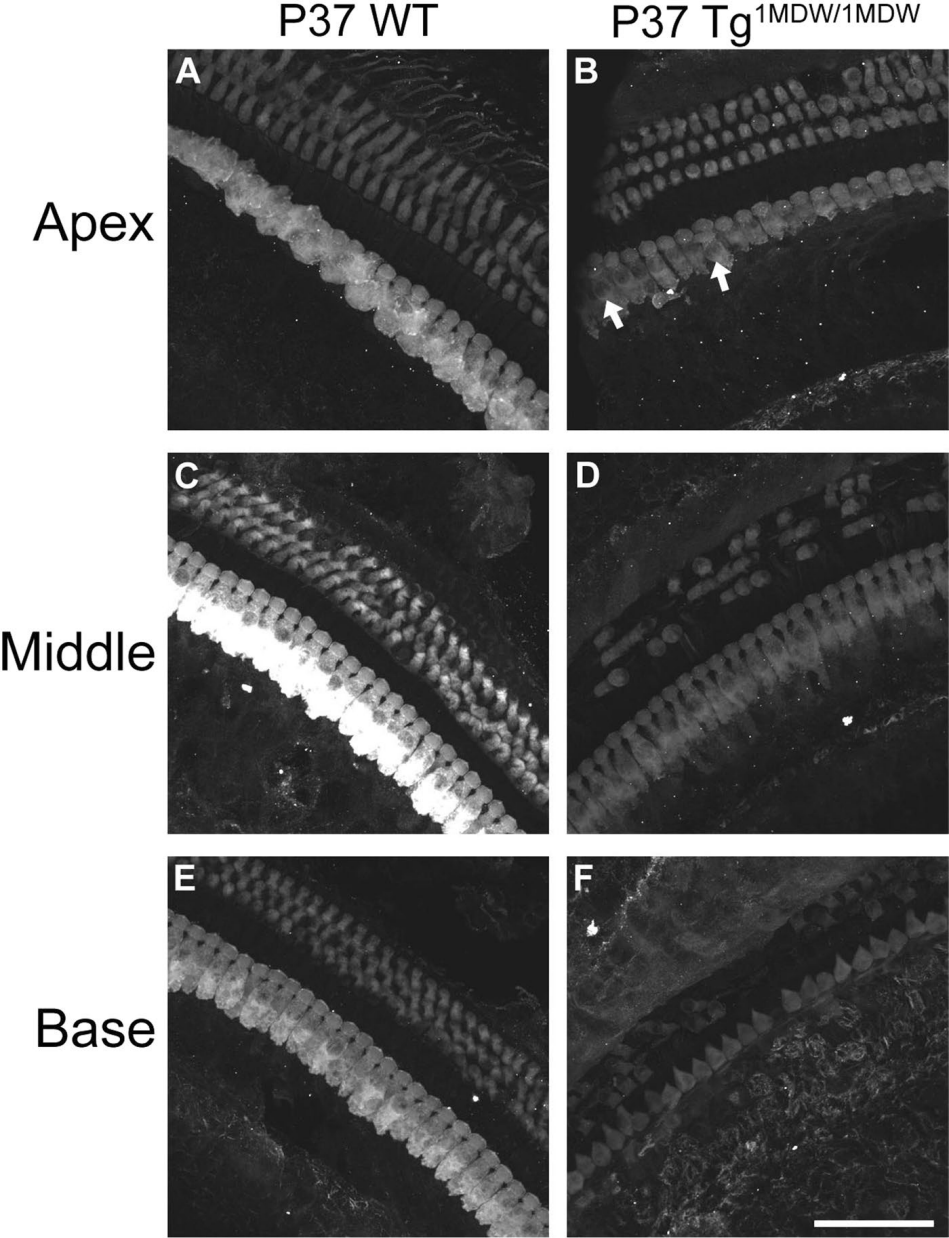
Gradients of HC loss and HC dismorphology in Tg^1MDW/1MDW^ at P37. Immunofluorescence of whole mount cochleae labeled with primary antibodies to MYO7a in P37 WT and Tg^1MDW/1MDW^ mice. The images are brightest point projections from Z series stacks parallel to the reticular lamina. Fields were imaged from comparable locations along the cochlear duct (**A**,**B** Apex, **C**,**D** Middle, **E**,**F** Base). Note the base to apex decrease in OHC loss with the concomitant increase in shortened and rounded OHCs. The presence of extra medially located IHCs in the Tg^1MDW/1MDW^ apex are also noted (B, white arrows). Scale bar; 50 μM.

Homozygous Tg^1MDW/1MDW^ mice lose Preyer’s reflex, an indication of hearing loss^24^. Consistent with that, we found evidence of OHC loss in the cochlear base at P18 (see Fig. 3). Moreover, phalloidin staining of cochlear whole mounts demonstrate missing OHCs in the basal OC at P21 (Fig. 7A–D) and stereocilia defects, which includes membrane fusion and bundle architecture defects at P30 (Fig. 7E,F). Immunofluorescence of whole mount cochleae at P37 labeled with primary antibodies to MYO7a confirm a base-to-apex degeneration of cochlear HCs (Fig. 8), with many in Tg^1MDW/1MDW^ OHCs altered from a cylindrical shape to being significantly shorter with larger diameter basal ends compared to WT OHC (Fig. 8). By P115, very few IHC or OHC remain (data not shown).

**Figure 9:**
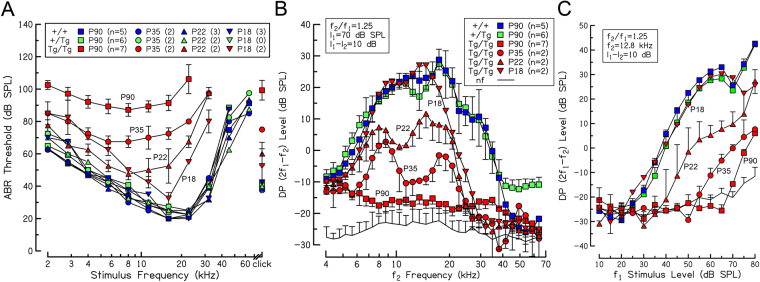
Hearing loss in Tg^1MDW/1MDW^ mice. (**A**) ABR threshold vs. frequency curves from P18 to P90. High-frequency (>16 kHz) hearing loss was observed in replicate P18 aged Tg^1MDW/1MDW^ homozygotes. At P22, high-frequency hearing loss of replicate homozygotes extended to frequencies above 8 kHz and by P35 hearing loss was observed across all test frequencies. By P90, the lowest thresholds observed in P90 Tg^1MDW/1MDW^ homozygotes were in the 90 dB SPL range, and responses were not detected at the highest level of output for stimulus frequencies >22.6 kHz, nor in homozygotes from P18 to P35 at frequencies >32 kHz. (**B**) Average distortion product otoacoustic emission (DPOAE) amplitude vs. f_2_ frequency curves confirm the sensory nature of hearing loss in homozygotes. DPOAEs observed in Tg^1MDW/1MDW^ homozygous mice were near the noise floor. (**C**) Average DPOAE input/output curves at an f_2_ frequency of 12.8 kHz indicate progressive DPOAE threshold shifts during the first 90 days.

Consistent with histological analyses, average auditory brainstem response (ABR) threshold (dB SPL) vs. frequency curves demonstrate progressive hearing loss from P18 to P90 in homozygous Tg^1MDW/1MDW^ mice (Fig. 9A). Responses were not detected even at the highest levels for stimulus frequencies >32 kHz between P18 and P35, nor were responses detected at frequencies >22.6 kHz in P90 Tg^1MDW/1MDW^ mice. A two-way mixed analysis of variance of ABR threshold as a function of stimulus frequency from animals at P90 yielded a significant genotype X stimulus interaction, F(16,120) = 30.89, *P* < 0.001, a significant main effect of genotype, F(2,15) = 1228.48, *P* < 0.001 and a significant main effect of stimulus, F(8,120) = 119.72, *P* < 0.001. Bonferroni corrected post-hoc tests showed significant threshold differences between WT and homozygous Tg^1MDW/1MDW^ animals (*P* < 0.001), and hemizygous and homozygous Tg^1MDW/1MDW^ animals (P < 0.001), but not between WT and hemizygous animals (*P* = 0.067). One-way ANOVAs showed significant differences among genotypes for all stimuli tested and multiple comparison tests indicated that thresholds of homozygous Tg^1MDW/1MDW^ mice were significantly higher than WT and hemizygous mice for all stimuli tested.

Average distortion product otoacoustic emission (DPOAE) amplitude frequency curves confirm the peripheral sensory nature of hearing loss in Tg^1MDW/1MDW^ mice (Fig. 9B). Although DPOAEs were observed at P90 in Tg^1MDW/1MDW^ mice, responses were near the noise floor. A two-way mixed ANOVA of DPOAE level as a function of f2 frequency from animals at P90 yielded a significant interaction between frequency and genotype, F(64,480) = 6.1 P < 0.001, a significant main effect of frequency, F(32,480) = 23.59, *P* < 0.001, and a significant main effect of genotype, F(2,15) = 9.9, *P* < 0.05 (*P* = 0.002). Bonferroni corrected post-hoc tests showed significant amplitude differences between WT and homozygous Tg^1MDW/1MDW^ animals (p = 0.01), as well as between hemizygous and homozygous Tg^1MDW/1MDW^ animals (*P* = 0.003) but not between amplitudes of WT and hemizygotes (*P* = 1.0). One-way ANOVAs indicated significant differences among genotypes for f_2_ frequencies from 7.3 kHz to 29.3 kHz and multiple comparison tests showed significant differences in DPOAE level between homozygous Tg^1MDW/1MDW^ mice and both WT and hemizygous mice, but not between WT and hemizygous mice.

Stimulus level-dependent, progressive hearing loss was also apparent as shown in a series of DP amplitude vs. stimulus level curves representing responses to an f2 frequency of 12.8 kHz (Fig. 9C). Progressively increasing DPOAE thresholds with age were observed, findings that are consistent with the observed inner ear pathology, and confirming that the hearing impairment reported here results from sensory HC degeneration. A 2-way mixed ANOVA of DPOAE level as a function of f1 level from animals at P90 yielded a significant interaction between level and genotype, F(26,169) = 35.9 *P* < 0.001, a significant main effect of level, F(13,169) = 178.9, *P* < 0.001, and a significant main effect of genotype, F(2,13) = 56.8, *P* < 0.001. Bonferroni corrected post-hoc tests showed significant amplitude differences between WT and homozygous Tg^1MDW/1MDW^ animals (*P* < 0.001), as well as between hemizygous and homozygous Tg^1MDW/1MDW^ animals (*P* < 0.001), but not between amplitudes of WT and hemizygotes (*P* = 1.0). One-way ANOVAs showed significant differences among genotypes at levels at and above 30 dB SPL and multiple comparison tests indicated significant differences between homozygous Tg^1MDW/1MDW^ and WT mice at levels >25 dB SPL and between homozygous Tg^1MDW/1MDW^ and hemizygous mice at levels >35 dB SPL, although, responses for homozygous Tg^1MDW/1MDW^ mice were at noise floor levels below 70 dB SPL.

Both ABR and DPOAE measures correlate with the degree of histological HC loss in Tg^1MDW/1MDW^ mice (Figs. 6, 7). Overall, sensory HC degeneration in Tg^1MDW/1MDW^ mice suggests a significant potency of these three miRNAs in effecting tissue homeostasis through GFAP promoter-driven miRNA-183 cluster misexpression and represents a novel biological reagent useful to identify molecular pathways and mRNAs targeted by the miR-183 cluster.

## Discussion

Transgenic Tg^1MDW/1MDW^ mice were developed to misexpress miR-183 cluster in OC SCs to further explore the role of these HC-specific miRNAs on OC cell differentiation. In WT OC, the cell-specific mutual exclusion of miR-183 cluster expression from their respective mRNA targets ensures that HCs enforce repression of genes both temporally and spatially, relative to those same genes in SCs. The mutual exclusion hypothesis of miRNA function^16^ predicts that in Tg^1MDW/1MDW^, miR-183 cluster misexpression, driven by the GFAP promoter in adjacent and lineage-related SCs (compare Fig. 1A,B) should repress evolutionarily conserved target genes that are simultaneously essential to SC function and incompatible for attaining and/or maintaining the HC fate. The majority of downregulated genes observed by microarray (Table 2) at P5 are glial cell specific, supporting a significant influence of miR-183 cluster gene regulation on glial cell misexpression, most likely from Schwann cells that underlie the greater epithelial ridge (GER) and inner sulcus in the P5 OC microdissected tissue. At P6, possible effects on OC cell differentiation in Tg^1MDW/1MDW^ were revealed by detecting a modest increase in medially placed IHCs (Fig. 2A–C), and by the observation of SC types (Deiters’ cells) that express the HC marker MYO6 (Fig. 3A,B). Whether these cells are the result of SC transdifferentiation and/or are generated through perturbations of cell-cell signaling pathways known to effect such changes, such as Wnt and Notch signaling^35^, is the focus of our ongoing studies. Previous studies with this cluster have shown them to be potent modulators of HC fates in zebrafish *in vivo*^11^. These extra IHCs phenocopy single-gene hypomorphic and null mutations in genes that specify SC identity and that are predicted targets of miR-183 cluster miRNAs (Fig. 2D)^25–29,36–38^. Indeed, our dual luciferase assays validated *Sox2*, *Notch1* and *Hes1* 3′UTRs as targets for post-transcriptional repression by miR-183 cluster members (Fig. 4). Cochlear HCs and adjacent SCs of the mammalian OC arise from post-mitotic prosensory precursors within specialized otic epithelium^20^. Notch1, Jagged1 (Jag1, a Notch ligand) and a downstream transcription factor (TF), Sox2, define this precursor pool through the Notch-mediated process of lateral induction^39^. Notch signaling also governs the process of lateral inhibition, which results in sharply contrasting cell types in the OC. In this process, presumptive HCs upregulate the TF Atoh1, notch ligands Delta1 (Dll1) and Jagged 2 (Jag2), and the miR-183 cluster. Dll1 and Jag2 increase notch1 activation in adjacent SC cells. This activation releases the notch intracellular fragment, N^ICD^, inducing genes that inhibit HC fates and promote SC fates^40^, including expression of GFAP, a glial-specific intermediate filament protein^41^. Interestingly, Sox2 persists in adult OC SCs, suggesting a maintenance of precursor identity^42^. Since Notch signaling is regulated by miRNAs in *Drosophila*^43^, one testable hypothesis using Tg^1MDW/1MDW^ is to ask whether SC miR-183 cluster expression affects Notch-mediated cell fate specification and/or homeostasis as the mechanism for how targeted misexpression of the HC specific miR-183 cluster may increase the propensity of SCs to transdifferentiate into HCs, an emerging paradigm for treating hearing loss^44–46^.

Rapid age-related demise of HCs, observed in this model both histologically (Figs 7, 8) and physiologically (Fig. 9), suggests a specific potency to dysregulation of miR-183 cluster target genes on postnatal OC homeostasis and function. While it can’t be ruled out that integration of Tg^1MDW^ may have disrupted an endogenous gene critical for hair cell survival, comparing Tg^1MDW/1MDW^ whole transcriptome effects to those from previously published miR-183 cluster mouse loss-of-function mutants (Fig. 6, Table 5) revealed reciprocal effects on miR-183 cluster target sites. These reciprocal effects are consistent with a reduction of miR-183 cluster miRNAs in sensory cells^2,33^ versus a gain of miR-183 cluster miRNAs in SCs (this study). Despite these fundamental miRNA target-specific transcriptome differences, sensory cell demise predominates in all three mouse models. Interestingly, comparing the 19,358 interrogated genes common to both P5 OC Tg^1MDW/1MDW^ versus P3 OC *Mir96*^*ddl/ddl*^ microarray studies, the overlap of *Mir96*^*ddl/ddl*^ changed genes (n = 97) with Tg^1MDW/1MDW^ changed (179) genes (uncorrected P < 0.05, fold change cutoff ± 1.5) were statistically not independent (Odds Ratio 7.27, Fisher Exact test P = 0.00028) with all 6 common genes changed in the same direction (5 downregulated: *Ncmap, Pmp22, Prx, Mag, Fa2h*, 1 upregulated: *Stfa1*). So, while there is evidence for reciprocal effects on miR-183 cluster target sites, these models do exhibit common gene expression changes, as well. Indeed, the common HC phenotypes in regards to stereocilia defects, ABR threshold elevation and HC death, while more severe in *Mir96*^*+/ddl*^ heterozygotes, are similar and suggest a narrow range of tolerance for modulations in miR-183 cluster miRNA levels, and therefore the genes they regulate.

This mammalian miRNA misexpression model demonstrates the potency of small non-protein coding miRNAs and should be useful in genomic/transcriptomic/proteomic studies to identify primary miR-183 cluster target genes and regulatory, structural and/or metabolic pathways affected by their dysregulation. The elucidation of miRNA-regulated pathways affected in SCs may provide novel avenues for future therapeutic interventions in treating some forms of hearing loss, be it by identifying molecular genetic pathways critical to HC homeostasis or by informing SC transdifferentiation research.

## Methods

### Animals

Animal studies were approved by both Creighton University and Boys Town National Research Hospital Institutional Animal Care and Use Committees and were consistent with the National Research Council *Guide for the Care and Use of Laboratory Animals* (2011). FVB/NCrl mice were purchased from Charles River Laboratories.

### Generation of transgenic mice and genotyping

A 453 bp DNA fragment encompassing the pre-miRNA sequences of both *miR183* and *miR96* were PCR amplified from total mouse DNA using the following primers: 5′-CAGTCCCGGGTGCAGGCTGGAGAGTGTGAC-3′ and 5′-GATCGATATCCCTCAGGCAGTGAAAGGTGATC-3′. A 586 bp region including pre-miR-182 was PCR amplified using primers 5′-CATGGATATCGGGCTTGAGGAGGTTTTACAC-3′ and 5′-GTACGCGGCCGCGATCGCATAGACCAGAAGACAC-3”. The miR-183/miR-96 PCR product was directionally cloned into the XmaI-EcoRV sites within the polycloning region of pIRES-hrGFPII (Stratagene) to create p183-X-E. The miR-182 PCR product was subsequently cloned into the EcoRV-NotI sites of p183-X-E to create p182-10. The cloned sequences for both plasmids were verified by DNA sequencing. HEK293 cells transfected with p182-10 verified expression and processing of significantly elevated levels of mature miR-183 cluster miRNAs using commercial miRNA PCR assays (Ambion, data not shown). To generate the miR-183 cluster transgene, a 1045 bp PCR product having *Mir183*, *Mir96* and *Mir182* was amplified from p182-10 using the following PCR primers: 5′-CAG TAG ATC TTG CAG GCT GGA GAG TGT GAC-3′ and 5′-CTG AAG ATC TGA TCG CAT AGA CCA GAA GAC AC-3. This PCR product was digested with BglII and replaced the nls-LacZ BamHI fragment of pGFA2-nlac (a gift from M. Brenner, UAB) to create pGfa2-miRs-183-96-182. A linear 3.8 kb NspI fragment from pGfa2-miRs-183-96-182 (genbank ID: JX912274) was purified and used for pronuclear microinjection into FVB/NCrl embryos. Three founders (Tg^1MDW^, Tg^2MDW^, Tg^3MDW^) were identified by PCR amplification of a 512 bp DNA product using the following primers: 5′-TTG GCA ATG GTA GAA CTC ACA C-3′ and 5′ATC TGC TCC TGC TTT TGC TG-3. Independent transgenic lines of the founders were established by backcrossing to FVB/NCrl and maintained by filial mating. Real-time quantitative PCR was used to genotype animals for genetic, molecular and histologic studies described below. Briefly, 5′ nuclease assay primer/probe sets for Tg(GFAP- *Mir183,Mir96,Mir182*) and endogenous *Atoh1* gene were simultaneously amplified in PCR reactions with Platinum® Quantitative PCR SuperMix-UDG w/ROX (Invitrogen) using DNA purified from tail biopsies. Table 6 lists the primer sequences used for quantitative Tg genotyping. PCR was run on a StepOnePlus™ PCR system (Applied Biosystems®). Tg genotypes were determined using the ΔΔCt method using *Atoh1* as the normalization control. The hemizygote and homozygote quantitative PCR distributions within each transgenic line were statistically distinct (Fig. 1).

### Histologic analysis

Whole-mount *in situ* hybridization (ISH) using LNA-DIG labeled probes against miR-182 was performed as previously described^7^. Cy5 fluorescence of miR-182 used sheep anti-DIG-POD Fab antibodies (Roche) with a tyramine signal amplification kit (TSA Plus, Perkin Elmer). For fluorescent labeling of F-actin, mouse cochleae were harvested, microdissected, fixed in 4% PFA overnight, dehydrated-rehydrated through an ethanol series, blocked with normal goat serum and stained overnight with TRITC-Phalloidin (Sigma, 0.5ug/ml), rinsed in PBS, mounted on slides with glycerol and imaged using a Zeiss LSM 510 META LNO confocal microscope. For electron microscopy, ears were fixed overnight in 2.5% glutaraldehyde, postfixed in 1% OsO4 in cacodylate buffer, dehydrated, sputter coated and mounted for scanning electron microscopy (SEM).

For immunohistochemistry, tissue pieces of the OC (apex, middle and base) were block/permeabilized with 5% Normal Goat Serum (NGS)/0.1% Tween20 at room temperature for 2–3 hours, incubated with polyclonal rabbit MYO6 or MYO7a primary antibodies (Proteus Biosciences, #25-6791-MYO6, #25-6790-MYO7a) for 48 hours in blocking buffer and washed three times with PBS, labeled with either Alexa 488- or Alexa-568- conjugated anti-rabbit secondary antibodies (1:500) (Invitrogen), washed with PBS, coverslipped using Prolong anti-fade (Invitrogen), and analyzed using either a Zeiss LMS 510 or LMS 800 confocal microscope.

### Real-time quantitative RT-PCR

Relative expression levels of mature miR-96, miR-82, miR-183 were assayed using ABI Taqman assays according to the ΔΔCt method using *snoRNA-202* (Supplementary Figure S1) or *snoRNA-135* as the normalization control.

### Dual luciferase assays

DNA corresponding to the 3′ UTRs of Sox2, Notch1, Jag1, and Hes1 mRNA was amplified from mouse genomic DNA by PCR. Products were purified and inserted into the region corresponding to the 3′ UTR of the Photinus luciferase reporter gene in pmirGLO (Promega) using the In-Fusion HD Cloning System (Clontech). pmirGLO also contains a separate Renilla luciferase report gene. HEK293 cells (~2 × 10^5^ cell/well; 24-well plate) were co-transfected using Lipofectamine 2000 (Invitrogen) with 200 ng reporter vector and 20 pmol synthetic RNA duplex representing scrambled control siRNA (Integrated DNA Technologies), miR-96, miR-182, or miR-183, or with 30 pmol combined miRNAs (10 pmol each). Cells were cultured post-transfection for 24 h and harvested to perform dual-luciferase assays using the Dual-Glo Luciferase Assay System (Promega) on a Modulus Microplate Luminometer with dual injectors (Turner Biosystems). Two replicate readings from six transfections over the course of three experiments were performed. The ratio of Photinus and Renilla luciferase activity for each reporter vector co-transfected with miRNA was normalized to that reporter vector co-transfected with scrambled control siRNA. The Wilcoxon signed-rank test was used to determine statistically significant differences in relative luciferase activity between miRNAs and control siRNA.

### MicroArray Study

Ear tissues were collected and stored in RNAlater at −80 °C. Total RNA from WT and Tg^1MDW/1MDW^ microdissected OC at P5 (n = 5) and cochleae at P18 (n = 3) were purified using the RNAeasy Mini Kit (Qiagen) following rotor-stator homogenization. The integrity and quality of each RNA preparation were determined using RNA pico chips run on a Model 2100 Agilent BioAnalyzer and quantitated using a NanoDrop 2000 spectrophotometer. RNA samples were sent to the University of Nebraska Medical Center (UNMC) microarray core facility for labeling and microarray hybridization to Affymetrix mouse MoGene 1.0 platform (Affymetrix). From a total of 28,188 annotated probesets, mean differences in gene expression were statistically identified by applying a 2-way ANOVA block design using the Linear Model for Microarray Data (Limma) Package^47,48^ through implementation of MicroArray Я US Gui interface to R^49^ and the Bioconductor Project microarray analysis programs. The microarray data has been assigned a GEO omnibus accession GSE95579.

### Cochlear function

Auditory brainstem responses (ABRs) and distortion product otoacoustic emissions (DPOAEs) were recorded in WT, Tg^1MDW^ hemizygous and Tg^1MDW/1MDW^ homozygous mice anesthetized with a ketamine-xylazine mixture (100 mg/kg ketamine, 15 mg/kg xylazine IP) and supplemented with 25–50% of the initial dose as needed throughout the recording session to maintain a stable, quiet recording environment. Body temperature was controlled using a thermostatically regulated heating blanket and thermal probe, and body temperature was maintained at approximately 37.5 °C (Harvard Apparatus). Heart rate was monitored throughout the procedure and fluids were replaced as needed. All recordings were conducted in an electrically shielded, double-walled, sound-attenuating chamber (Industrial Acoustics Corp).

### ABR Procedures

ABRs were used to assess the integrity of the cochlea and auditory brainstem non-invasively as detailed previously^50,51^. Stimuli consisted of 3 ms pure tone bursts (1 ms raised cosine on/off ramps and 1 ms plateau) or 64μs clicks. Both tone bursts and clicks were digitally generated (125 kHz clock rate) and delivered free-field through a high impedance piezoelectric tweeter (Radio Shack). For stimuli above 32 kHz, an electrostatic speaker (ES1, TDT) was substituted. Sound sources were placed ~10 cm from the cranial vertex. Stimulus levels were calibrated in decibels sound pressure level (dB SPL) with a 1/8-inch Brüel and Kjær microphone (Model 4138). Platinum needle electrodes (Grass Instruments) were positioned subdermally at the vertex (active, non-inverting), the infra-auricular region (reference, inverting), and the neck region (ground). Scalp voltage potentials were amplified 100,000X, band-pass filtered between 0.03 and 10 kHz (Grass Model P511K), and digitized (Tucker-Davis Technologies, TDT) at a 20 kHz sampling rate over 15 milliseconds. A total of 200 trials were averaged for each response. Waveforms were stored digitally for off-line analyses and custom software was used for data acquisition and subsequent data analyses. ABR thresholds were determined for clicks and for tone bursts in half octave steps ranging from 64 to 2.0 kHz. Stimulus levels were decremented from 90 dB SPL to below threshold in 10 dB steps. Threshold values were subsequently refined using a bracketing strategy in which level was adjusted in 5 dB steps relative to threshold values previously determined in 10 dB step decrements. Threshold was defined as the smallest stimulus that generated an unambiguous, replicable response.

### DPOAE Procedures

DPOAEs were used to assess OHC function non-invasively as detailed previously^51,52^. Briefly, 2 phase-locked pure tone stimuli (f1, f2) were generated by 24-bit D/A converters (Lynx22 soundcard) and conveyed through separate earphones. The primary tone frequencies were presented such that f2/f1 = 1.25, and f2 intensity was 10 dB lower than f1. The earphone outputs, along with a low-distortion probe microphone (Etymotic Research, ER-10B+) were sealed within the external ear canal, forming a closed acoustic system. Acoustic emissions recorded by the probe microphone were amplified 40 dB and sampled with a 24-bit A/D converter (Lynx Studio Technology, L22) at 48 kHz for tone frequencies below 5 kHz or at 192 kHz at tone frequencies above 5 kHz. FFT analyses were used to compute level/phase of component DPOAEs and their corresponding noise floors.

Physiological results were analyzed using a two-way mixed analysis of variance (IBM SPSS version 22), with genotype (WT, Tg^1MDW^ hemizygous and Tg^1MDW/1MDW^ homozygous groups) as the between-subject variable. For ABRs, stimulus type (click and frequencies from 2 to 22.6 kHz) was the repeated measure, for DPOAE frequency sweeps, f2 frequency (4 to 64 kHz) was the repeated measure, and for DPOAE input-output curves, f1 level (10 to 75 dB SPL) was the repeated measure. Bonferroni adjustments were made for multiple comparisons and data were analyzed further using one-way analysis of variance (ANOVA) and Dunnett C tests were used for multiple comparisons. Differences between means were considered statistically significant when *P* < 0.05.

## Electronic supplementary material

Supplementary Figure S1
