# Supplementary material for: A mouse model of miR-96, miR-182 and miR-183 misexpression implicates miRNAs in cochlear cell fate and homeostasis
Source: Sci Rep. 2018 Feb 23;8:3569. doi: 10.1038/s41598-018-21811-1 (PMC5824881; doi:10.1038/s41598-018-21811-1)
Supplement: Supplementary file 1 — Supplementary Figure S1 [file 41598_2018_21811_MOESM1_ESM.pdf]

A mouse model of miR-96, miR-182 and miR-183 misexpression implicates miRNAs in cochlear cell fate and homeostasis.

Authors: Michael D. Weston[1*], Shikha Tarang[1], Marsha L. Pierce[2], Umesh Pyakurel[1], Sonia Rocha-Sanchez[1], JoAnn McGee[3], Edward J. Walsh[3], Garrett A. Soukup[4]

Addresses:

1. School of Dentistry, Department of Oral Biology, Creighton University 780729 California Plaza, Omaha NE 68178-0729
2. School of Medicine, Department of Pharmacology, Creighton University 2500 California Plaza , Omaha NE 68178
3. Developmental Auditory Physiology Laboratory, Boys Town National Research Hospital, 555 North 30th Street, Omaha NE 68131
4. School of Medicine, Department of Biomedical Sciences, Creighton University 2500 California Plaza, Omaha NE 68178

*Corresponding author:

Michael D. Weston, Ph.D.
Creighton University
School of Dentistry
Boyne Building
Department of Oral Biology
780729 California Plaza
Omaha, NE 68178-0729
Phone: 402-280-5030
email: michaelweston@creighton.edu

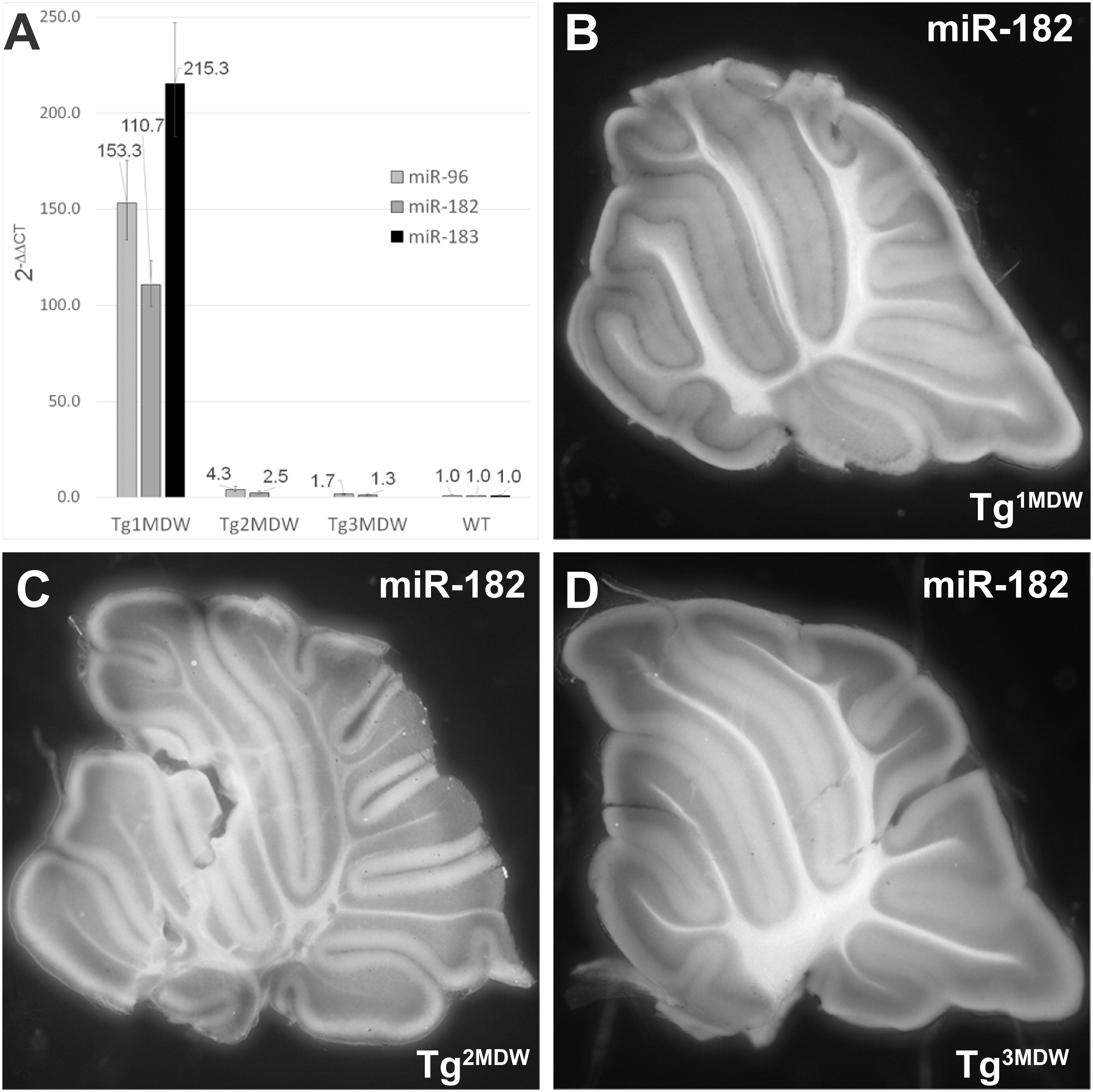
A
250.0
200.0
150.0
100.0
50.0
0.0
2-ΔΔCT
153.3
110.7
215.3
miR-96
miR-182
miR-183
4.3
2.5
1.7
1.3
1.0 1.0 1.0
Tg1MDW
Tg2MDW
Tg3MDW
WT
B
miR-182
Tg1MDW
C
miR-182
Tg2MDW
D
miR-182
Tg3MDW

Supplementary Figure S1. Three FVB/NClr-Tg(GFAP-Mir183,Mir96,Mir182) lines demonstrate variable patterns and levels of miR-183 cluster miRNA expression. (A). TaqMan assays for miR-96, miR-182 and miR-183 relative expression ($log2^{-\Delta\Delta CT}$, normalized to *snoRNA-202* and WT littermates of Tg^{3MDW} or Tg^{1MDW}) from total brain RNA of Tg obligate hemizygotes (P21), reveals the variability of expression/abundance. Tg^{1MDW} levels of miR-183 cluster miRNAs are substantially higher than either Tg^{2MDW} or Tg^{3MDW} and are likely caused by transgene integration position effects. (B-D). *In situ* detection of miR-182 in parasagittal cerebellar slices from weaned Tg hemizygotes. (B). Tg^{1MDW} shows miR-182 localization consistent with Bergmann glial cells demonstrating the GFAP core promoter expression in endogenous GFAP expressing cells. (C-D). Tg^{2MDW} and Tg^{3MDW} do not demonstrate the same cerebellar expression pattern as Tg^{1MDW}. The pattern and level differences of miR-183 cluster miRNAs in the brain are consistent with other phenotypic distinctions between these lines (i.e. lens cataracts, pelage loss, hearing loss) which are most likely due to transgene copy number and integration site differences.
